# Hrs and SNX3 Functions in Sorting and Membrane Invagination within Multivesicular Bodies 

**DOI:** 10.1371/journal.pbio.0060214

**Published:** 2008-09-02

**Authors:** Véronique Pons, Pierre-Philippe Luyet, Etienne Morel, Laurence Abrami, F. Gisou van der Goot, Robert G Parton, Jean Gruenberg

**Affiliations:** 1 Department of Biochemistry, University of Geneva, Geneva, Switzerland; 2 Institute of Global Health, Ecole Polytechnique Fédérale de Lausanne, Lausanne, Switzerland; 3 Institute for Molecular Bioscience and Centre for Microscopy and Microanalysis, University of Queensland, Brisbane, Australia; Stanford University, United States of America

## Abstract

After internalization, ubiquitinated signaling receptors are delivered to early endosomes. There, they are sorted and incorporated into the intralumenal invaginations of nascent multivesicular bodies, which function as transport intermediates to late endosomes. Receptor sorting is achieved by Hrs—an adaptor-like protein that binds membrane PtdIns3P via a FYVE motif—and then by ESCRT complexes, which presumably also mediate the invagination process. Eventually, intralumenal vesicles are delivered to lysosomes, leading to the notion that EGF receptor sorting into multivesicular bodies mediates lysosomal targeting. Here, we report that Hrs is essential for lysosomal targeting but dispensable for multivesicular body biogenesis and transport to late endosomes. By contrast, we find that the PtdIns3P-binding protein SNX3 is required for multivesicular body formation, but not for EGF receptor degradation. PtdIns3P thus controls the complementary functions of Hrs and SNX3 in sorting and multivesicular body biogenesis.

## Author Summary

The cell's genetic program is modulated by extracellular signals that activate cell surface receptors and, in turn, intracellular effectors, to regulate transcription. For cells to function normally, these signals must be turned off to avoid permanent activation—a situation often associated with cancer. For many receptors, signaling is repressed, or down-regulated, in a process that first internalizes and then degrades the receptors. After receptors are removed from the cell surface into structures called early endosomes, they are selectively incorporated within vesicles that form inside the endosome. During this process, endosomal membranes are pulled away from the cytoplasm towards the endosome lumen, against the flow of intracellular membrane traffic, eventually resulting in the formation of a “multivesicular body” (vesicles within vesicles). The common view is that these intralumenal vesicles are then delivered to lysosomes, where they are degraded along with their receptor cargo. We have investigated the mechanisms responsible for the biogenesis of intralumenal vesicles in multivesicular bodies. We find that the small protein SNX3, which binds the signaling lipid phosphatidyl inositol-3-phosphate, is necessary for the formation of intralumenal vesicles, but is not involved in the degradation of the cell surface receptor for EGF. Conversely, we find that Hrs, which also binds phosphatidyl inositol-3-phosphate and mediates receptor sorting into intralumenal vesicles, is essential for lysosomal targeting but dispensable for multivesicular body biogenesis. Phosphatidyl inositol-3-phosphate thus controls the complementary functions of Hrs and SNX3 in the sorting of signaling receptors and multivesicular body biogenesis.

## Abstract 3

SNX3 plays a direct role in the formation of intralumenal vesicles of multivesicular bodies (MVBs) but is not involved in EGF receptor degradation, whereas Hrs is essential for lysosomal targeting but dispensable for MVB biogenesis. Hence, intralumenal vesicle formation in MVB biogenesis can be uncoupled from lysosomal targeting.

## Introduction

Cell surface lipids and proteins as well as solutes are endocytosed into animal cells through several routes, including clathrin-coated pits and vesicles, caveolae, and pathways that do not depend on caveolae or clathrin [1,2]. Some receptors may be differentially sorted into clathrin-coated pits or caveolae/rafts, depending on their fate: signaling or degradation [3–5]. These endocytic routes all seem to lead to early endosomes eventually; from which some components, including housekeeping receptors, are returned to the cell surface for reutilization while others are transported to the trans-Golgi network and the biosynthetic pathway. By contrast, signaling receptors that need to be down-regulated, including the activated EGF receptor, as well as other endocytosed proteins and lipids, are efficiently sorted away from recycling molecules within early endosomes, and are then routed towards late endosomes and lysosomes, where degradation occurs [6,7].

Major progress has been made in understanding the molecular mechanisms responsible for EGF receptor sorting in early endosomes. Sorting signals are provided by the addition of multiple ubiquitin molecules to the receptor cytoplasmic domain [8]. Ubiquitin coupled to the receptor then binds Hrs through its ubiquitin interacting motif, while Hrs also interacts simultaneously with membrane PtdIns3P via a FYVE domain and with clathrin, leading to the concentration of activated receptor molecules into specialized clathrin-coated regions of early endosomes [9–11]. The epidermal growth factor (EGF) receptor then interacts sequentially with the ESCRT-I, ESCRT-II, and ESCRT-III protein complexes, and eventually appears within lumenal vesicles present in multivesicular regions of the early endosomes [12–14]—a process that uncouples activated receptors from cytosolic signaling partners and thus efficiently terminates signaling. This mechanism also appears to contribute to the ubiquitin-independent sorting of the delta opioid G-protein–coupled receptor in mammalian cells [15] and Sna3p in yeast [16–18]. However, sorting of the melanosomal protein Pmel17 does not depend on ubiquitin, Hrs, and ESCRT components [19], and likewise, other proteins, including proteins of the limiting membrane, are presumably transported in an ESCRT-independent manner.

Eventually, these multivesicular regions detach—or mature—from early endosomes, and become free multivesicular endosomes or bodies (MVBs), which then serve as transport intermediates (ECVs or endosomal carrier vesicles), in the microtubule-dependent transport towards late endosomes—herein referred as ECV/MVBs. Formation of ECV/MVBs appears to be itself under the control of EGF signaling [20] and depends on annexin family members [21,22], whereas conversion of the small GTPase Rab5 to Rab7 may occur on early endosome [23] or during early-to-late endosome transport [24]. Upon fusion with late endosomes, ECV/MVB lumenal vesicles are delivered to late endosomes, and are eventually packaged within lysosomes, where they are degraded together with their cargo of down-regulated receptors.

Evidence shows that the sorting process mediated by Hrs and ESCRTs is coupled somehow to the mechanism of membrane invagination towards the lumen of the endosome, including during the topologically equivalent process of HIV budding at the plasma membrane, which takes advantage of the ESCRT machinery [25–28]. In yeast, mutation of the genes that encode ESCRTs and other components of this pathway leads to the formation of an aberrant endosomal compartment (Class E VPS phenotype). Then, endocytosed membrane proteins that would normally be degraded accumulate in this Class E compartment but also on the membrane of the vacuole (see [12] and reference therein), which is functionally equivalent to lysosomes. Similarly, Hrs knockout in *Drosophila* [29] or knockdown in mammalian cells [20,30] causes a reduction in the number of lumenal vesicles, presumably because the downstream machinery responsible for membrane invagination fails to assemble in cells lacking Hrs. Knockdown of Tsg101, an ESCRT-I subunit, causes pleiotropic changes in early endosome morphology, including tubulation, reduction in the number of lumenal vesicles [20], and perhaps, the formation of a mammalian equivalent of the yeast Class E compartment [31]. While it is clear that Hrs and ESCRTs play an essential role in the lysosomal targeting of many ubiquitinated proteins, the mechanism controlling membrane invagination itself remains elusive. All proteins that are known to induce membrane deformation act in the topologically opposite direction—towards the cytoplasm—via direct insertion into the bilayer or protein–lipid interactions [32].

Here, we have further investigated the mechanisms that control endosomal membrane dynamics, and in particular the role of PtdIns3P and its effectors. Indeed, PtdIns3P is well known to regulate endocytic membrane traffic and protein sorting through numerous effectors that contain FYVE or PX PtdIns3P-binding domains. Moreover, PtdIns3P controls both the sorting of signaling receptors [33] and the biogenesis of lumenal membranes [34–36] at least in part via the FYVE-containing protein Hrs [37]. Searching for other PtdIns3P effectors, we screened proteins of the sorting nexin (SNX) family that all contain the phosphoinositide-binding PX domain [38]. Several SNX proteins play a role in protein trafficking and some contain a BAR domain, involved in sensing and/or inducing membrane curvature [39]. We find that SNX3 plays a direct role in multivesicular body formation, but is not involved in EGF receptor degradation. By contrast, Hrs seems to be essential for lysosomal targeting but dispensable for multivesicular body biogenesis and transport to late endosomes.

## Results

**Figure 1:**
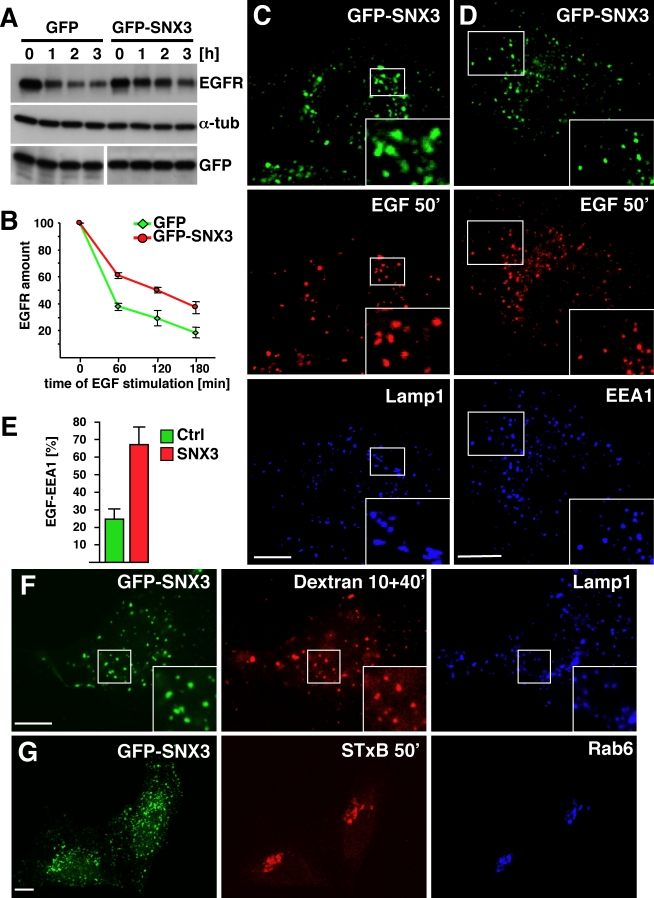
SNX3 in Endosomal Transport (A and B) HeLa cells expressing GFP-SNX3 were incubated with 0.25 μg/ml EGF and 10 μg/ml cycloheximide for the indicated time periods. Cell lysates (100 μg) were analyzed by SDS gel electrophoresis and western blotting with antibodies against EGFR (20-ESO4 against a peptide of the cytoplasmic domain), α-tubulin (α-tub), or GFP. Blots were scanned and the quantification is shown in (B). (C and D) After cell surface binding, biotin-EGF coupled to streptavidin-R-phycoerythrin was internalized for 50 min at 37 °C in HeLa cells expressing GFP-SNX3. Cells were labeled with antibodies against Lamp1 (C) or EEA1 (D) and analyzed by triple channel fluorescence. The various combinations of merged colors for (C) and (D) are shown in Figure S3A and S3B, respectively. Insets in the lower right are a magnification of the regions shown in the boxes. (E) Individual endosomes containing both EGF and EEA1 were counted in (D) and in control cells (Figure S4A). Values are expressed as a percentage of the total number of EGF-containing endosomes (>10 cells per experiment). (F) Rhodamine-dextran was pulsed for 10 min at 37 °C in HeLa cells expressing GFP-SNX3 and then chased for 40 min. Cells were analyzed as in (C and D) using antibodies against Lamp1, and the various combinations of merged colors are in Figure S3C. (G) After cell surface binding, Shiga toxin B-subunit conjugated to Cy3 was internalized for 50 min at 37 °C into HeLa cells expressing GFP-SNX3. Cells were analyzed as in (C and D) using antibodies against Rab6, and the various combinations of merged colors are the same as in Figure S3D. In (B and E), each condition is the mean of three independent experiments; standard errors are indicated. (C, D, F, and G) Scale bar indicates 10 μm.

When ectopically expressed, green fluorescent protein (GFP)-tagged SNX3 showed a characteristic punctate distribution, and colocalized with EEA1, an effector of the small GTPase Rab5, and to some extent with the transferrin receptor, but not with late endocytic markers, including lysobisphosphatidic acid (LBPA) (Figure S1A) or Lamp1 (Figure 1C and 1F). Consistently, GFP-SNX3 cofractionated with Rab5, but not with LBPA (Figure S1B). GFP-SNX3 was thus primarily present on early endosomes, much like the endogenous protein [40], and this association depended on PtdIns3P and required an intact PtdIns3P-binding domain PX (Figure S1C), as expected [40].

To investigate the possible role of SNX3, we first tested whether overexpression affected transport along the endocytic pathway. SNX3 overexpression had no effect on EGF receptor endocytosis, since internalized EGF colocalized with both GFP-SNX3 and EEA1 in early endosomes within 10 min of incubation at 37 °C (Figure S2A), much like in control cells (unpublished data, see [33]). However, upon longer incubation times at 37 °C, EGF receptor degradation was delayed by SNX3 overexpression (Figure 1A; quantification in Figure 1B), in agreement with previous findings [40]. This delay was caused by defective transport, since the bulk of endocytosed EGF failed to reach late endosomes containing Lamp1 (Figures 1C and S3A) or LBPA (unpublished data) after 50 min in cells overexpressing SNX3. Then, EGF remained in early endosomes containing GFP-SNX3 and EEA1 (Figures 1D and S3B; quantification in Figure 1E), whereas it was mostly transported beyond early endosomes in control cells (Figure S4A). The inhibitory effect of SNX3 was specific, since overexpression of other SNX family members, including SNX1, SNX2, and SNX16, did not interfere with EGF receptor transport (Figure S5), as expected [41,42].

Much like with EGF receptor, endocytosis of the bulk phase marker rhodamine-dextran was not affected by SNX3 overexpression, and the tracer accumulated in early endosomes containing GFP-SNX3 and EEA1 within 10 min at 37 °C (Figure S2B). However, after a subsequent 40-min chase at 37 °C, SNX3 overexpression markedly impaired dextran transport to late endosomes. The bulk of the tracer then remained in early endosomes and failed to reach Lamp1-positive late endocytic compartments (Figures 1F and S3C), as was observed with the EGF receptor (Figure 1C and S3A) and in contrast to control cells (Figure S4B). These inhibitory effects of excess SNX3 were specific, since SNX3 overexpression did not affect the transport of endocytosed Shiga toxin B-subunit from early endosome (Figure S2C) to the trans-Golgi network (Figures 1G and S3D; compare with controls in Figure S4C), which requires SNX1 [43]. Altogether these observations indicate that SNX3 overexpression caused the selective retention of signaling receptors and bulk tracers in early endosomes containing Rab5 and its effector EEA1, and thereby inhibited more-distal steps of early-to-late endosome transport.

**Figure 2:**
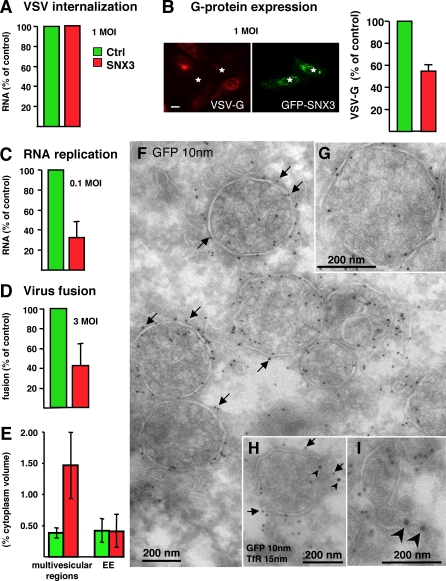
SNX3 in VSV Infection and MVB Biogenesis (A) VSV (1 multiplicity of infection [MOI]) was bound on ice to the surface of control HeLa cells (ctrl) or HeLa cells expressing GFP-SNX3. Cells were incubated for 15 min at 37 °C to allow VSV internalization. Then, total endosomes were prepared by fractionation and viral RNA was quantified by RT-PCR. Values are expressed as a percentage of untransfected controls. (B) Experiments were as in (A), except that cells were incubated for 3 h, instead of 15 min, at 37 °C to allow VSV infection to proceed. Cells were analyzed by immunofluorescence with antibodies against VSV-G protein (left panels); stars indicate cells expressing GFP-SNX3 and not VSV-G. The total number of cells expressing the G-protein was counted and is expressed as a percentage of the controls (≈60% of untransfected control cells were infected). (C) Experiments were as in (B), except that 0.1 MOI VSV was used and viral RNA replication was quantified by RT-PCR. Values are expressed as a percentage of untransfected controls. (D) Experiments were as in (A), except that 3 MOI Dil-labeled VSV was used and that cells were incubated for 35 min at 37 °C. Then, viral fusion events were visualized as fluorescent spots (due to Dil dequenching) by fluorescence microscopy, and quantified. Values are expressed as in (B). (E) Control HeLa cells (Figure S6A) or cells expressing GFP-SNX3 (Figure S6B) were fixed and embedded in Epon. Random fields of the different areas in one section were captured at 25.000× magnification (the procedure was repeated 3× for controls and 5× after GFP-SNX3 overexpression). Early endosomes (EE) were identified as vacuole of 200–500 nm containing five or fewer clearly defined internal vesicles, and multivesicular regions as vacuole of 200–500 nm containing five or more clearly defined spherical internal vesicles [66]. The volume of EE and multivesicular regions was calculated by stereological means, and the mean values are expressed as a percentage of the cytoplasm volume ± standard error of the mean (SEM). (F–I) HeLa cells expressing GFP-SNX3 were analyzed by electron microscopy after immunogold labeling of cryosections using antibodies against GFP ([F–H], arrows) and the transferrin receptor (TfR) ([H and I], arrowheads) followed by proteinA-gold, as indicated. In (A–E), each condition is the mean of at least three independent experiments; standard errors are indicated. (B) Scale bar indicates 10 μm; (F–I) scale bar indicates 0.2 μm.

To further investigate the role of SNX3 in early-to-late endosome transport, we made use of vesicular stomatitis virus (VSV), which infects cells from the endocytic pathway. Indeed, endocytosed virions must be transported beyond early endosomes for efficient infection to occur [44,45]. Overexpression of SNX3 had no effect on endocytosis of VSV (Figure 2A), as observed with EGF and dextran (Figure S2A and S2B), but significantly reduced VSV infection, which was monitored by the synthesis of the viral glycoprotein G (Figure 2B). This inhibition did not result from some indirect effects of SNX3 on the G-protein biosynthetic pathway, since replication of the viral genome, quantified by real time-PCR (RT-PCR), was also similarly reduced (Figure 2C), indicating that excess SNX3 prevented efficient release of the viral nucleocapsid into the cytosol.

The viral nucleocapsid is released into the cytoplasm after low pH-triggered fusion of the viral envelope with endosomal membranes. To monitor viral fusion events, VSV was labeled with self-quenching amounts of Dil, a fluorescent long-chain dialkylcarbocyanine dye, and bound to the cell surface at 4 °C [44]. After endocytosis at 37 °C, the fusion of individual virions was revealed in the light microscope by the appearance of fluorescent spots in endosomes, due to Dil dequenching [44]. Strikingly, overexpression of SNX3 markedly reduced VSV fusion (Figure 2D). Since VSV fusion normally occurs beyond early endosomes [44,45], these observations indicate that virions, much like EGF receptor and dextran (Figure 1) remained trapped in early endosomes in cells overexpressing SNX3.

**Figure 3:**
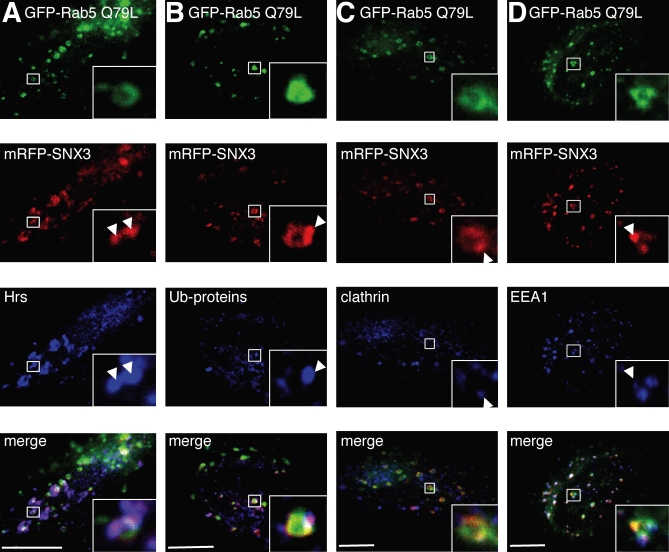
SNX3 Colocalizes with Hrs, Ubiquitinated Proteins, and Clathrin on Rab5^Q79L^ Enlarged Endosomes. (A–D) HeLa cells co-expressing both GFP-Rab5^Q79L^ and mRFP-SNX3 were processed for immunofluorescence using different antibodies as indicated and analyzed by triple channel fluorescence. Arrowheads point at regions containing GFP-SNX3. Scale bar indicates 10 μm. Insets in the lower right are a magnification of the regions shown in the boxes.

The effects of SNX3 overexpression on the transport of viral particles, EGF receptor, and fluid phase markers led us to investigate by electron microscopy whether endosome morphology was then affected. SNX3 overexpression caused a dramatic accumulation of multivesicular structures without causing a general expansion of endosome vesicular regions (Figure S6; quantification in Figure 2E), perhaps suggesting that only specialized regions of the early endosome are competent to become MVBs. Multivesicular elements after SNX3 overexpression were frequently clustered in groups of five to ten individuals, as revealed in low (Figure S6) and high (Figure 2F) magnification views, perhaps connected to each other (Figure 2F). Immunogold labeling of cryosections showed that GFP-SNX3 itself was abundant on the limiting membrane of these multivesicular structures. These SNX3-positive structures all exhibited a similar multivesicular and spherical appearance (diameter ≈ 0.4 μm), which closely resembles the morphology of MVBs or ECVs that mediate early-to-late endosome transport [46]—herein referred to as ECV/MVBs. However, in marked contrast to free ECV/MVBs during transport towards late endosomes, these SNX3-positive structures exhibited the characteristic features of early endosomes, including all early endosomal markers that were tested (Figure 1 and see Figure 3). Moreover, the transferrin receptor, which is restricted to early and recycling endosomes, was found closely associated with these SNX3-positive ECV/MVB-like structures (arrowheads in Figure 2H and 2I), consistent with data from us (Figure 1) and others [40]. The transferrin receptor was often present in tubulo-cisternal elements connected to multivesicular elements (Figure 2I), presumably corresponding to forming recycling endosomes. It thus appears that SNX3 overexpression causes the accumulation of multivesicular regions on early endosomal membranes. These ECV/MVB-like structures fail to detach, or to mature, from early endosomes. Such a frustrated process of ECV/MVB formation accounts nicely for our observations that the transport of EGF receptor, VSV and bulk markers beyond early endosomes is then inhibited (see model, Figure S10).

In our electron microscopy analysis, SNX3 was often observed on, or close to, membrane regions containing electron-dense materials on the cytoplasmic membrane face (e.g., Figure 2G), which resembled the early endosomal Hrs-clathrin coat that mediates ubiquitinated receptor sorting into lumenal vesicles during ECV/MVB formation on early endosomes [9,10]. To further characterize SNX3 distribution, we thus used the constitutively active mutant Rab5^Q79L^, which induces the formation of enlarged early endosomes by promoting their homotypic fusion. On these large endosomes, regions containing Hrs, clathrin, and ubiquitinated receptors could be resolved by light microscopy from those containing EEA1, indicating that components of the machinery that sorts down-regulated receptors into ECV/MVB internal vesicles are concentrated in specific early endosomal domains [9]. When GFP-Rab5^Q79L^ was coexpressed with monomeric red fluorescent protein (mRFP)-SNX3, both proteins were present on enlarged early endosomes, as expected. Approximately 90% of endosomes containing SNX3 were also labeled with Rab5^Q79L^, but some variation in SNX3 association with individual Rab5^Q79L^-endosomes was observed (Figure 3), presumably reflecting different angles of visualization in the confocal planes. SNX3 was clearly seen to colocalize preferentially with Hrs (Figure 3A), ubiquitinated proteins (Figure 3B), and clathrin (Figure 3C) in regions that seemed devoid of EEA1 (Figure 3D). These observations further demonstrate that ectopically expressed SNX3 accumulates on early endosomal membranes. They also indicate that this accumulation occurs preferentially in multivesicular regions that contain, in addition to SNX3 itself, the protein machinery responsible for sorting into lumenal vesicles. It thus seems that the lumenal invagination process continues in the presence of excess SNX3, leading to the accumulation of multivesicular regions on early endosomes, but that more distal transport events, including ECV/MVB detachment—or maturation—(Figures 1A–1F and 2B–2D), are inhibited, perhaps because excess SNX3 limits the access or binding of downstream machineries.

**Figure 4:**
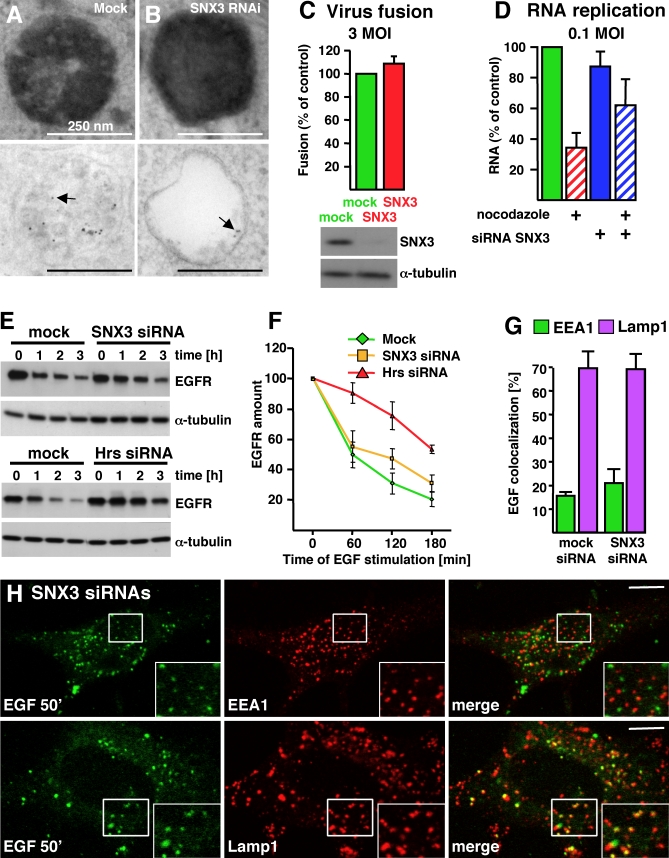
SNX3 Silencing Inhibits Membrane Formation within MVBs but Does Not Affect EGFR Early-to-Late Endosomal Transport and Degradation (A) The ECV/MVB content of mock-treated HeLa cells was labeled with endocytosed HRP [22,46]. Samples were processed for plastic embedding after HRP cytochemical detection, and analyzed by electron microscopy. The micrographs show representative high-magnification views of individual HRP-containing structures (upper panel). In separate experiments, the ECV/MVB content was labeled with 5-nm proteinA-gold endocytosed for 30 min at 37 °C (lower panel). (B) As in (A), but cells were treated with SNX3 siRNAs. (C) SNX3 siRNA or mock-treated HeLa cells were incubated with 3 MOI Dil-labeled VSV and viral fusion was monitored as in Figure 2D (inset: SNX3 down-regulation in cells treated with SNX3 siRNAs). (D) HeLa cells were treated with SNX3 siRNAs or mock-treated, and then microtubules were depolymerized or not with 10 μM nocodazole. Then, cells were infected with 0.1 MOI VSV, and RNA replication was quantified as in Figure 2C. (E and F) HeLa cells treated with SNX3 siRNAs, Hrs siRNAs, or mock-treated were stimulated with EGF and analyzed as in Figure 1A. Blots were scanned and the quantification is shown in (F). (G) Individual endosomes containing both EGF and EEA1 or Lamp1 were counted in SNX3 siRNA-treated cells (see [H] below) and in control cells (Figure S8A). Values are expressed as a percentage of the total number of EGF-containing endosomes in each category (>10 cells per experiment). (H) EGF-biotin coupled to streptavidin-AlexaFluor 488 was endocytosed as in Figure 1C and 1D in cells treated with SNX3 siRNA. Cells were analyzed by immunofluorescence with the indicated antibodies. Insets in the lower right are a magnification of the regions shown in the boxes. In (C and D) and (F and G), each condition is the mean of at least three independent experiments; standard errors are indicated. (A and B) Scale bar indicates 0.25 μm; (H) scale bar indicates 10 μm.

**Figure 5:**
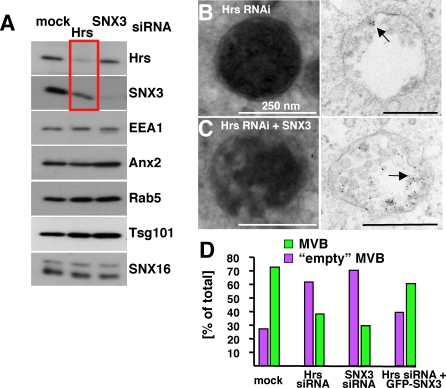
SNX3 Rescues the Formation of Internal Vesicles in Hrs siRNA-Treated Cells (A) HeLa cells treated with siRNAs against Hrs or SNX3 or mock-treated were lysed. Lysates were analyzed by SDS gel electrophoresis and western blotting using indicated antibodies. The red box highlights the reduction in SNX3 levels observed after Hrs knockdown. (B) HeLa cells were treated with Hrs siRNAs. Then, the endosomal content was labeled with HRP (left panel) or 5-nm proteinA-gold (right panel) and analyzed by electron microscopy as in Figure 4A. (C) As (B), except that GFP-SNX3 was overexpressed during the last 24 h. (D) The number of MVBs and “empty” MVBs were counted in the experiments shown in Figure 4A (mock-treated cells), Figure 4B (SNX3 siRNAs), Figure 5B (Hrs siRNAs), and Figure 5C (Hrs siRNAs followed by SNX3 overexpression), and are expressed as a percentage of the total number of HRP-labeled endosomes (≈50 individual endosomes counted for each condition). All structures containing one or more intralumenal vesicles were counted as MVBs). To ensure unbiased analysis and quantification, micrographs were taken in Brisbane, and each condition was number coded. Analysis and quantification were then performed blind in Geneva.

**Figure 6:**
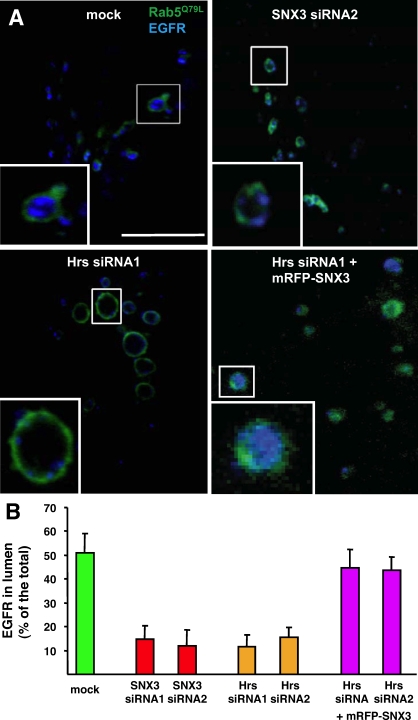
SNX3 Controls the Formation of Intralumenal Vesicles That Incorporate the EGF Receptor (A and B) HeLa cells were mock-treated or treated with siRNAs against SNX3 or Hrs (two different siRNAs in each case) and transfected with GFP-Rab5^Q79L^ (green) during the last 24 h. Alternatively, mRFP-SNX3 was overexpressed in cells treated with each anti-Hrs siRNA. After cell surface binding, EGF was internalized for 15 min at 37 °C. Cells were labeled with anti-EGF-R antibodies (blue) and analyzed by confocal microscopy. In (B), the relative amount of EGF-R in the lumen of endosome was quantified [50] and expressed as the percentage of the total amount of EGF-R. Each condition is the mean of at least three independent experiments; standard errors are indicated. (A) Scale bar indicates 10 μm. Insets in the lower left are a magnification of the regions shown in the boxes.

Since overexpression of SNX3 caused an expansion of multivesicular regions on early endosomes, we investigated the impact of SNX3 down-expression on the formation of lumenal vesicles by electron microscopy. To this end, the lumen of ECV/MVBs was labeled with endocytosed horseradish peroxidase (HRP) pulsed for 15 min and then chased for 30 min at 37 °C, after microtubule depolymerization with 10 μM nocodazole [46]. As expected [46], multivesicular structures with the characteristic ECV/MVB morphology were found in controls (Figure 4A, upper panel), accounting for approximately 70% of the total HRP-positive structures (see quantification in Figure 5D). By contrast, after SNX3 knockdown to approximately 20% of the control levels (inset in Figure 4C), approximately 70% of the total HRP-positive profiles did not seem to contain lumenal vesicles, but were otherwise similar to controls (Figure 4B, upper panel). All SNX3 knockdown experiments were repeated with two small interfering RNA (siRNA) target sequences without significant differences (see Figures 6A, 6B, and S8C).

To better visualize the presence or absence of lumenal vesicles, ECV/MVBs were labeled with 5-nm proteinA-gold endocytosed for 30 min at 37 °C with or without nocodazole. In the mock-treated control, the gold particles distributed within ECV/MVBs, which typically contained approximately 20 vesicles per profile, whether microtubules were present (Figure 4A, lower panel) or not (unpublished data). After SNX3 knockdown, however, 5-nm gold particles labeled vesicles of the same diameter as ECV/MVBs, but with only three to five internal vesicles per profile, whether microtubules were intact (Figure 4B, lower panel) or not (unpublished data). The appearance and size of internal vesicles were otherwise undistinguishable from the controls. Altogether, our data thus indicate that SNX3 plays a direct and specific role in the formation of intralumenal membrane invaginations within nascent ECV/MVBs, since multivesicular regions are increased by overexpression (Figure 2F) and decreased by down-expression (Figure 4B).

Interestingly, SNX3 knockdown did not affect virus fusion (Figure 4C) and caused only a small, marginal decrease in nucleocapsid release (Figure 4D). Previously, we had found that the VSV envelope undergoes fusion primarily with the membrane of ECV/MVB internal vesicles, thus releasing the capsid into their lumen, where it remains hidden [44]. In late endosomes, back fusion of these vesicles with the endosome-limiting membrane then ensures capsid delivery to the cytoplasm, indicating that VSV fusion and capsid release occur in sequential steps of the pathway. In particular, we found that depolymerization of the microtubules, which reduces early-to-late endosome transport [46], does not affect viral fusion, but efficiently inhibits VSV delivery to late endosomes and capsid release [44] (see Figure 4D). In contrast to controls, capsid release was only marginally affected by microtubule depolymerization in cells treated with SNX3 siRNAs (Figure 4D)—much like in cells treated with PI 3-kinase inhibitors or Hrs siRNAs [44], which both decrease intralumenal membranes in endosomes [30,35] (see also Figure 5B). This was not due to some indirect effects of SNX3 siRNAs, since early-to-late endosome transport remained microtubule dependent after SNX3 knockdown (see below and Figure S7A and S7B). It thus appears that, when ECV/MVBs lack intralumenal vesicles after SNX3 knockdown, VSV fusion can be triggered at the limiting membrane, thus by-passing the need for transport to late endosomes—again much like after PI 3-kinase inhibition or Hrs knockdown [44].

The “empty” endosomes in cells lacking SNX3 closely resemble endosomes observed after Hrs knockdown in mammalian cells [30] (see Figure 5B) or mutagenesis in *Drosophila* [29]. In these studies, Hrs depletion also inhibited EGF receptor degradation, supporting the view that sorting into intralumenal invaginations mediates lysosomal targeting [13,37]. To our surprise, SNX3 knockdown had little effect on EGF receptor degradation (Figure S7C), significantly less than Hrs knockdown (see blot in Figure 5A) in parallel experiments (Figure 4E, quantification of the blots in Figure 4F). Consistently, a wave of fluorescent EGF reached late endocytic compartments containing Lamp1 in cells treated with SNX3 siRNAs (Figure 4H; quantification in Figure 4G) as in mock-treated cells (Figure S8A), and this transport required intact microtubules (Figure S7A and S7B). Then, EGF no longer colocalized with EEA1 in early endosomes (Figure 4H; quantification in Figure 4G) as in mock-treated cells (Figure S8A). This is in contrast to the inhibition observed after SNX3 overexpression (Figure 1C–1E). SNX3 knockdown thus appears to prevent the formation of intralumenal invaginations within endosomes without interfering with EGF receptor transport and degradation, indicating that the lysosomal targeting of signaling receptors is then uncoupled from sorting into ECV/MVBs.

Both Hrs and SNX3 seem to play a role in the membrane invagination process, but only Hrs, and not SNX3, appears to be involved in EGF receptor targeting to lysosomes, perhaps suggesting that Hrs acts upstream of SNX3 in receptor sorting and multivesicular body biogenesis. Consistent with this notion, Hrs knockdown selectively reduced the expression of SNX3—without affecting any other protein involved in endosome membrane dynamics that we tested (Figure 5A)—and in particular decreased the membrane-associated pool of SNX3 (Figure S8B). By contrast, SNX3 knockdown had no effect on Hrs expression (Figure 5A and see Figure S8C). We thus wondered whether the known effect of Hrs knockdown could be due, at least in part, to reduced levels of SNX3.

As expected [30], approximately 60% of the total HRP-labeled endosomes appeared to contain fewer internal membranes (Figure 5B, left panel; quantification in Figure 5D) in cells treated with Hrs siRNAs, much like endosomes in cells treated with SNX3 siRNAs (Figure 4B)—and in contrast to endosomes in mock-treated cells (Figure 4A). Parallel analysis of cells after proteinA-gold internalization revealed that these endosomes contained five to ten internal vesicles per profile, which were otherwise similar to controls (Figure 4A), whether microtubules were intact (Figure 5B, right panel) or not (unpublished data)—again much like after SNX3 knockdown and in contrast to controls (Figure 4A and 4B). The multivesicular morphology of endosomes in cells treated with Hrs siRNAs could, however, be restored by overexpression of GFP-SNX3 (Figure 5C; quantification in Figure 5D), despite that fact that Hrs expression remained silenced (Figure S8D). Structures labeled with 5-nm proteinA-gold then contained approximately 25–30 lumenal vesicles per profile, similar to endosomes in mock-treated controls. These data thus strongly suggest that the morphological phenotype of endosomes in Hrs-depleted cells is at least in part due to low SNX3 levels, and also demonstrate that SNX3 is a component of the molecular machinery that drives intralumenal membrane invagination.

It has been suggested that EGF receptor is trafficked through a subpopulation of multivesicular endosomes in a process that involves annexin A1 [21]. Annexin A1 is structurally, functionally, and biochemically related to annexin A2 [47], and both proteins colocalize on early endosomes [48,49]. Annexin A2 was also proposed to play a role in multivesicular endosome biogenesis, but not in the invagination process [22]. Consistently, endogenous annexin A1 colocalized with annexin A2-GFP and mRFP-SNX3, and endogenous annexin A2 with mRFP-SNX3 (Figure S9A). These observations show that SNX3 is present on endosomes that contain both annexin A1 and annexin A2.

Next, we investigated whether SNX3 plays a role in the formation of intralumenal vesicles that mediate EGF receptor sorting into multivesicular endosomes. To this end, we made use of the ability of the active Rab5 mutant Rab5Q79L to form enlarged early endosomes that provide high spatial resolution by light microscopy [9,50], as in Figure 3. When mock-treated cells were challenged with EGF for 15 min at 37 °C, the EGF receptor was endocytosed into these enlarged endosomes, where greater than 50% of the endocytosed receptor accumulated in the lumen (Figure 6A, quantification in Figure 6B), as expected [50]. Similarly, EGF colocalized with the receptor in the lumen of these enlarged endosomes (unpublished data). Knockdown of Hrs with either one of two siRNAs significantly reduced EGF receptor sorting into the lumen of enlarged endosomes (Figure 6A, quantification in Figure 6B), consistent with our electron microscopy analysis (Figure 5B–5D) and in agreement with previous findings [30,50]. Similarly, SNX3 depletion with either one of two siRNAs inhibited EGF receptor incorporation in the lumen of large endosomes to the same extent as Hrs knockdown (Figure 6A, quantification in Figure 6B). Finally, SNX3 re-expression in the Hrs knockdown background restored EGF receptor accumulation in the lumen of enlarged endosomes to the same extent as observed in mock-treated controls (Figure 6A, quantification in Figure 6B). These observations unambiguously demonstrate that SNX3 controls the formation of lumenal membranes that carry the EGF receptor, further confirming the role of SNX3 in the lumenal invagination process.

**Figure 7:**
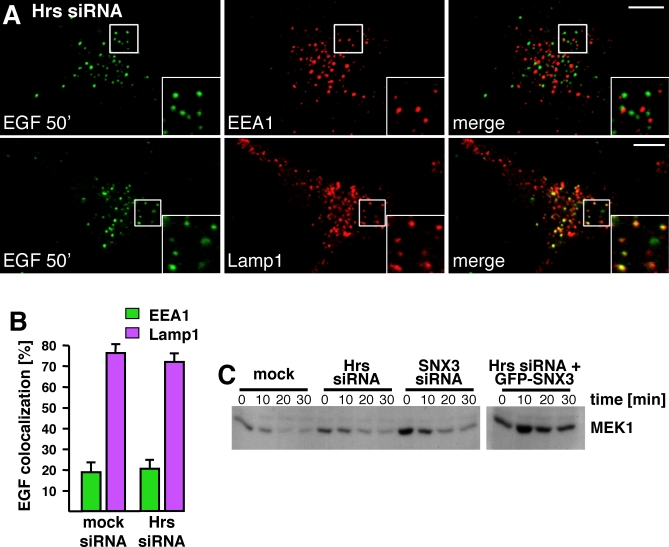
MEK1 Cleavage by Anthrax Toxin Lethal Factor (A and B) Experiments (A) and quantification (B) were as in Figure 4G and 4H, except that cells were treated with Hrs siRNAs. Insets in the lower right are a magnification of the regions shown in the boxes. (C) Hrs or SNX3 was knocked down, or Hrs was knocked down and GFP-SNX3 overexpressed. The trypsin-nicked Protective Antigen (500 ng/ml) and the Lethal Factor (100 ng/ml) of anthrax toxin were then bound to the cell surface on ice [51], and cells were incubated at 37 °C. Lysates (40 μg) were analyzed by western blotting using antibodies against MEK1 N-terminus to detect MEK1 cleavage by Lethal Factor released into the cytosol. In (B), each condition is the mean of at least three independent experiments; standard errors are indicated. (A) Scale bar indicates 10 μm.

Although reduced levels of SNX3 seemed to account for the invagination defect in Hrs knockdown cells, SNX3 siRNAs did not affect EGF receptor transport to late endosomes (Figure 4G–4H) and degradation (Figure 4E and 4F). Similarly, a wave of endocytosed EGF receptor was exported from EEA1-positive early endosomes and reached Lamp1-positive late endosomes (Figure 7A, quantification in Figure 7B) under our conditions of Hrs knockdown (≈80%, Figure 5A) much like in mock-treated cells (Figure S8A) or in cells treated with SNX3 siRNAs (Figure 4H). Since, under the same Hrs knockdown conditions, the formation of internal vesicles (Figures 5B and 6A) and EGF receptor degradation were inhibited (Figure 4E) and SNX3 levels reduced (Figure 5A), these observations strongly suggest that Hrs is an essential component of the lysosome targeting machinery, which can function independently of receptor sorting into and incorporation within multivesicular endosomes.

To further discriminate between Hrs and SNX3 functions, we made use of anthrax toxin, which is translocated across the membrane of ECV/MVB intralumenal vesicles. Like VSV nucleocapsids, the toxin hijacks these vesicles to reach late endosomes, where back fusion with the limiting membrane releases the lethal factor into the cytosol, leading to the cleavage of mitogen-activated protein kinase kinases (MAPKKs), and in particular MEK1 [51]. After addition of anthrax toxin, MEK1 cleavage was slightly retarded in cells treated with Hrs or SNX3 siRNAs (Figure 7C). Presumably, in the absence of intralumenal membranes (Figures 4B, 5B–5D, 6A, and 6B), toxin translocation could then occur across the limiting membrane of these “empty” ECV/MVBs—in good agreement with our observations on VSV capsid release after Hrs [44] or SNX3 (Figure 4D) knockdown. Interestingly, re-expression of GFP-SNX3 in the Hrs knockdown background prevented toxin translocation (Figure 7C). Presumably, the toxin, once released, remained trapped in the lumen of intralumenal vesicles. Similarly, EGF receptor degradation remained inhibited after re-expression of GFP-SNX3 in the Hrs knockdown background (Figure S9B). Indeed, excess SNX3 not only restored intralumenal vesicles (Figures 5C, 6A, and 6B), but also inhibited ECV/MVB detachment (or maturation), and thus transport beyond early endosomes towards late endosomes (Figures 1 and 2).

## Discussion

It is generally believed that the lysosomal targeting of signaling receptors is controlled by sorting via Hrs and its downstream ESCRT partners into membrane invaginations on early endosomal regions, which will become multivesicular endosomes. Then, intralumenal vesicles are transported to lysosomes, where they are degraded together with their receptor cargo. Our observations now demonstrate that lysosomal targeting can function independently of this multivesicular endosome sorting event, since the transport of EGF receptor to late endocytic compartments and its degradation are not affected by SNX3 knockdown, in the absence of ECV/MVB lumenal membranes (see model in Figure S10). This agrees with previous observations that PI 3-kinase inhibition with wortmannin inhibits the formation of lumenal vesicles, but not the delivery of the EGF receptor to the lysosomes [35]. A corollary of these observations, however, is that Hrs indeed controls lysosome targeting, as expected [29,30,52], but that this function can be uncoupled from the invagination process in the multivesicular pathway towards late endosomes. Even in the absence of ECV/MVB lumenal membranes after SNX3 knockdown, sorting into putative Hrs platforms [53] is sufficient to ensure, not only receptor transport to late endosomes, but also packaging into lysosomes and degradation.

Whereas Hrs is necessary for lysosomal targeting, the protein appears to be dispensable for the membrane invagination process that leads to ECV/MVB biogenesis. By contrast, SNX3, which is dispensable for lysosomal targeting, appears to function as a necessary component of the molecular machinery that drives the formation of membrane invaginations within forming ECV/MVBs. Indeed, SNX3 knockdown inhibits the invagination process, whereas excess SNX3 promotes the formation of intralumenal membranes and the accumulation of multivesicular regions on early endosomes. It thus appears that PtdIns3P signaling regulates differentially lysosomal targeting and the biogenesis of ECV/MVBs, via at least two PtdIns3P effectors, Hrs and SNX3, which act sequentially in the pathway. Although our data demonstrate that SNX3 regulates the membrane invagination process itself, the molecular mechanism is unclear. In yeast, the SNX3 homolog Grd19p is involved in selective retrieval of some membrane proteins from the prevacuolar compartment, presumably late endosomes, to the TGN [54,55]. It is not known whether the function of yeast Grd19p is somehow related to the function we propose for SNX3 in mammalian cells. However, it is possible that Grd19p and SNX3 play different roles, since endosome-to-Golgi transport of Shiga toxin B-subunit seems to require not SNX3 (this study), but SNX1 [43].

Several proteins and lipids were reported to play a role in the formation of intralumenal membranes, including Hrs and ESCRTs [13,14], SNX3 (this study), ceramide [50], and perhaps, LBPA and Alix [56]. Future work will be needed to determine the precise relationships that may exist between these components. At present, our observations indicate that SNX3 functions downstream of Hrs, and one may speculate that it recruits other proteins, perhaps ESCRTs, which then drive the invagination process. However, SNX3 is a short member of this protein family that does not contain any structural feature other than the PX domain itself [38,40]. Moreover, we failed to detect ESCRT components in SNX3 pull-downs, and two-hybrid screens showed that Grd19p interacts with proteins of the early secretory pathway, but the significance of these observations is not clear [57]. Alternatively, SNX3 itself may play a direct role in the invagination process by deforming membranes in a direction opposite to BAR-containing proteins [39].

## Materials and Methods

### Cells, antibodies, reagents, and constructs.

Cell maintenance was described [46], as was the mouse monoclonal anti-LBPA antibody [58]. We are very grateful to Ludger Johannes (Paris. France) for supplying us with Cy3-labeled Shiga toxin B-subunit; to Reinhard Jahn (Göttingen. Germany) and Volker Gerke (Muenster. Germany) for mouse monoclonal antibodies against RAB5 and annexin A2 (HH7), respectively; to Wanjin Hong (Singapore. Singapore), Harald Stenmark (Oslo, Norway), and Marino Zerial (Dresden, Germany) for rabbit polyclonal antibodies against SNX3 or SNX16, Hrs, and EEA1, respectively. We also used mouse monoclonal antibodies against: GFP (Roche Diagnostics); transferrin receptor (Zymed Laboratories); EEA1 and annexin A1 (Transduction Laboratories); human LAMP1 (Pharmacia); EGF receptor (BD Biosciences); α-tubulin (Sigma-Aldrich); ubiquitinated proteins (FK2) (Affinity Research Products); clathrin (X22) (ABR-Affinity BioReagents); and TSG101 (GeneTex). Polyclonal antibodies made in rabbit against Rab6 were from Santa Cruz Biotechnology and made in sheep against the EGF receptor from Fitzergald. HRP-labeled secondary antibodies were from Amersham or Sigma-Aldrich and fluorescently labeled secondary antibodies from Jackson Immunoresearch Laboratories. Wortmannin, nocodazole, EGF, HRP, and *o*-dianisidine were from Sigma-Aldrich, EGF-biotin, streptavidin R-phycoerythrin conjugate, AlexaFluor 488 EGF complex, 10-kDa rhodamine dextran from Molecular Probes. ProteinA-gold (5 nm) was from Utrecht University. We obtained pGFP-Rab5^Q79L^ from Marino Zerial (Dresden. Germany), GFP-tagged SNX1 and Flag-tagged SNX2 from Gordon N. Gill (University of California, San Diego, California), myc-tagged SNX3 from Carol R. Haft (National Institutes of Health, Bethesda, Maryland), and pDMYC-SNX16 from Wanjin Hong (Singapore, Singapore). SNX1, SNX2, and SNX3 were amplified by PCR and introduced into pEGFP-C2 or fused with mRFP.

### Viral fusion, RNA replication, and infection.

In experiments using VSV, the fusion of the viral envelope with endosomal membranes, the replication of viral RNA, and the appearance of the viral glycoprotein G in the host-cell biosynthetic pathway were analyzed as described [44]. Briefly, VSV fusion was measured using virus labeled with self-quenching amounts of the fluorescent dye Dil (long-chain dialkylcarbocyanine). Labeled virions were bound to the plasma membrane on ice and then cells were incubated for 35 min at 37 °C. Individual VSV fusion events resulted in the appearance of brightly fluorescent spots upon dye dequenching. For quantification, cells containing fused virions were counted. The replication of VSV RNA minus-strand was quantified by TaqMan RT-PCR; total RNA was extracted from infected HeLa cells, precipitated, and used for retrotranscription using specific oligonucleotides of the genomic VSV-G RNA. Finally, G-protein appearance in biosynthetic membranes 3 h postinfection was visualized by immunofluorescence using anti-VSV-G antibodies.

Endocytic transport in vivo. Cells were transfected with FuGene (Roche Diagnostics) according to the manufacturer's instructions. In RNA interference (RNAi) experiments, cells were transfected twice at a 24-h interval using OligoFectamin (Invitrogen) with 21-nucleotide RNA duplexes (Qiagen), replated 4 h after the second transfection, and analyzed 36 h later. In HeLa cells, we used an Hrs target sequence siRNA1 that was described [30] and already used in our previous studies [44], as well the target sequence siRNA2 5′-AAGCGGAGGGAAAGGCCACTT-3′; the target SNX3 sequences were: siRNA1, 5′-AAGGGCTGGAGCAGTTTATAA-3′; siRNA2, 5′-AACAAGGGCTGGAGCAGTTTA-3′. To follow fluid phase transport, cells were incubated for 10 min at 37 °C with 2.5 mg/ml rhodamine-dextran (pulse) and further incubated for 40 min without the marker (chase) when indicated. Alternatively, cells were incubated for 1 h at 4 °C with 1 μM Cy3-labeled Shiga toxin B-subunit, or incubated for 1 h at 4 °C with 400 ng/ml EGF-biotin-streptavidin-R-phycoerythrin complex or 500 ng/ml EGF-biotin-streptavidin-AlexaFluor488 complex. Then, the marker was endocytosed for the indicated time periods at 37 °C. To quantify EGF receptor degradation, cells were preincubated in serum-free medium, and incubated with 0.25 μg/ml EGF and 10 μg/ml cycloheximide for the indicated time periods. Microtubules were depolymerized with 10 μM nocodazole for 2 h [46,59]. When indicated, cells were incubated for 1 h at 4 °C with 500 ng/ml trypsin-nicked Protective Antigen (PA) and 100 ng/ml Lethal Factor (LF) of anthrax toxin, and then transferred to 37 °C for different periods of time in a toxin-free medium, as described [51].

### Microscopy.

Immunofluorescence microscopy has been described [60]. For annexin immunolabeling, cells were permeabilized before fixation with 100 mM KCl, 2 mM MgCl_2_, 1 mM CaCl_2_, and 1 mM Hepes (pH 6.9) containing 0.1% triton X-100 [61]. In some experiments, 500 ng/ml EGF-biotin-streptavidin-AlexaFluor488 complex was bound for 1 h at 4 °C to the surface of cells overexpressing Rab5Q79L, and then the complex was endocytosed for 15 min at 37 °C. The quantification of EGF receptor distribution in the lumen or on the limiting membrane of enlarged endosomes has been described [50]. Pictures were captured using a Zeiss Axiophot microscope equipped with a 63× Plan-NOEFLUAR objective or a Zeiss LSM 510 META confocal microscope equipped with a 63× Plan-Apochromat objective. To visualize the content of ECV/MVBs by electron microscopy, HRP was endocytosed for 15 min at 37 °C followed by a 30-min chase after microtubule depolymerization [22,46]. Cells were then fixed, HRP was revealed cytochemically with DAB as substrate, and processed for plastic embedding [62]. Alternatively, cells were incubated with 5-nm proteinA-gold (optical density [OD] at 520 nm = 10) for 30 min with or without microtubule depolymerization to label ECV/MVBs, and samples were processed as above. In this analysis, it was not possible to identify unambiguously cells that have been transfected. However, given the extent of knockdown observed on western blots (80%–90%), the corresponding protein (Hrs or SNX3) must be depleted in at least four out of five (or nine out of ten) cells, if depletion were complete in most cells, rather than partial in all cells. The latter view is consistent with observations that 100% of the cells were labeled with fluorescently labeled siRNAs (unpublished data). In any case, to ensure unbiased electron microscopy analysis and quantification of both HRP and proteinA-gold experiments, samples were prepared in Geneva and analyzed by western blotting to ensure that knockdown and re-expression of SNX3 were efficient. Then, samples were shipped to Brisbane. There, each experiment was number coded and analyzed by electron microscopy. The corresponding micrographs in number-coded folders were then transferred to Geneva. Analysis and quantification were then performed blind in Geneva. Electron microscopy after immunogold labeling of cryosections has been described [63].

### Other methods.

LBPA quantification by ELISA [58] was described, as was early and late endosome fractionation by flotation in a sucrose step gradient [59].

## Supporting Information

Figure S1 GFP-SNX3 Is Present on Early Endosomes and This Distribution Depends on PtdInsI3P (A) HeLa cells expressing GFP-SNX3 were processed for immunofluorescence using the indicated antibodies.
(B) BHK cells expressing GFP-SNX3 were homogenized and a postnuclear supernatant (PNS) was prepared. From this PNS, early endosomes (EE) were separated from late endosomes (LE) and heavy membranes (HM) by floatation in a sucrose step gradient [59,64]. Fractions were analyzed by SDS gel electrophoresis and western blotting, using antibodies against GFP to detect GFP-SNX3 or against the early endosomal marker Rab5 (upper panel), and by ELISA using antibodies against the late endosomal marker LBPA (lower panel).
(C) HeLa cells expressing GFP-SNX3 or the 3-phosphoinositide-binding defective mutant GFP-SNX3^R70A^ were treated or not with 100 nM wortmannin for 30 min at 37 °C and then analyzed by fluorescence microscopy. SNX3^R70A^ was no longer membrane-associated, much like the SNX3 R69RY to AAA triple mutant and Y71A single mutant [40].
(A and C) Scale bar indicates 10 μm.
(516 KB PDF)

Figure S2 The Internalization of EGFR, Dextran, and Shiga Toxin B-Subunit Is Not Affected in Cells Expressing GFP-SNX3 (A) HeLa cells expressing GFP-SNX3 were incubated with biotin-EGF coupled with streptavidin-R-phycoerythrin for 1 h at 4 °C, chased for 10 min at 37 °C, and analyzed by fluorescence microscopy after labeling with antibodies against EEA1.
(B) HeLa cells expressing GFP-SNX3 were incubated with rhodamine-dextran for 10 min at 37 °C, and analyzed as in (A).
(C) HeLa cells expressing GFP-SNX3 were incubated with 1 μM Shiga toxin B-subunit conjugated to Cy3 for 1 h at 4 °C, chased for 10 min at 37 °C, and analyzed by fluorescence microscopy after labeling with antibodies against the transferrin receptor.
(A–C) Scale bar indicates 10 μm.
(703 KB PDF)

Figure S3 SNX3 in Endosomal Transport (A) After cell surface binding, biotin-EGF coupled to streptavidin-R-phycoerythrin was internalized for 50 min at 37 °C in HeLa cells expressing GFP-SNX3. Cells were labeled with antibodies against Lamp1 and analyzed by triple channel fluorescence; the various combinations of merged colors are shown for the micrographs in Figure 1C.
(B) The experiment was as in (A), but cells were labeled with antibodies against EEA1. The various combinations of merged colors are shown for the micrographs in Figure 1D.
(C) Rhodamine-dextran was pulsed for 10 min at 37 °C in HeLa cells expressing GFP-SNX3 and then chased for 40 min. Cells were labeled with antibodies against Lamp1. The various combinations of merged colors are shown for the micrographs in Figure 1F.
(D) After cell surface binding, Shiga toxin B-subunit conjugated to Cy3 was internalized for 50 min at 37 °C into HeLa cells expressing GFP-SNX3, and cells were analyzed using antibodies against Rab6. The various combinations of merged colors are shown for the micrographs in Figure 1G.
(A–D) Scale bar indicates 10 μm
(1.76 MB PDF)

Figure S4 EGFR, Dextran, and Shiga Toxin B-Subunit Transport in Control Cells (A) HeLa cells were incubated with biotin-EGF coupled with streptavidin-R-phycoerythrin for 1 h at 4 °C and then chased for 50 min at 37 °C. Cells were then labeled with antibodies against EEA1 and analyzed by fluorescence microscopy.
(B) HeLa cells were incubated with rhodamine-dextran for 10 min at 37 °C, chased for 40 min, and then analyzed by fluorescence microscopy after labeling with antibodies against Lamp1.
(C) HeLa cells were incubated with 1 μM Shiga toxin B-subunit conjugated to Cy3 for 1 h at 4 °C and then chased for 50 min at 37 °C. Cells were then labeled with antibodies against Rab6 and analyzed by fluorescence microscopy.
(A–C) Scale bar indicates 10 μm.
(722 KB PDF)

Figure S5 Overexpression of SNX1, SNX2, or SNX16 Does Not Affect Transport of the EGF Receptor to Late Endosomes and Lysosomes HeLa cells expressing GFP-SNX1 (A), GFP-SNX2 (B), or myc-SNX16 (C) were incubated with biotin-EGF coupled with streptavidin-R-phycoerythrin for 1 h at 4 °C and then chased for 50 min at 37 °C. Cells were then labeled with antibodies against Lamp1 (A–C) or myc (C) and analyzed by fluorescence microscopy.
(A–C) Scale bar indicates 10 μm.
(535 KB PDF)

Figure S6 GFP-SNX3 Expression Causes an Expansion of the Multivesicular Regions of Early Endosomes Control HeLa cells (A) or HeLa cells expressing GFP-SNX3 (B) were fixed, embedded in Epon, and then processed for electron microscopy. Arrows point at structures with a characteristic multivesicular appearance. In (B), arrowheads surround a group of seven or eight multivesicular structures—such clusters were frequently observed in cells overexpressing GFP-SNX3 (quantification is shown in Figure 2E). Scale bar indicates 1 μm.
(403 KB PDF)

Figure S7 Early-to-Late Endosomal Transport Is Not Affected in Cells Treated with SNX3 siRNAs, and Depends on an Intact Microtubule Network (A and B) HeLa cells were treated with SNX3 siRNAs, and then microtubules were depolymerized (B) or not (A) with 10 μM nocodazole for 2 h. EGF-biotin coupled to streptavidin-AlexaFluor 488 was endocytosed for 50 min at 37 °C, and cells were analyzed by immunofluorescence using antibodies against Lamp1. Scale bar indicates 10 μm.
(C) HeLa cells expressing GFP-SNX3 were incubated with 0.25 μg/ml EGF and 10 μg/ml cycloheximide for the indicated time periods, as in Figures 1A and 4E. Cell lysates (100 μg) were analyzed by SDS gel electrophoresis and western blotting with an antibody raised against the purified recombinant partial cytoplasmic domain of human EGFR, as in Figures 1A and 4E. A blot of the complete gel is shown to illustrate the fact that degradation intermediates were not detected with this antibody. The arrow points at a nonspecific band.
(555 KB PDF)

Figure S8 Treatment with siRNAs (A) HeLa cells treated with mock siRNAs were incubated with 500 ng/ml EGF-biotin coupled with streptavidin-AlexaFluor 488 for 1 h at 4 °C, chased for 50 min at 37 °C, and analyzed by fluorescence microscopy after labeling with antibodies against EEA1 or Lamp1, as indicated. The quantification of these experiments is shown in Figure 4G. Scale bar indicates 10 μm.
(B) HeLa cells were mock-treated or treated with siRNAs against Hrs or SNX3 for 72 h. After homogenization, postnuclear supernatants were prepared and fractionated by high-speed centrifugation. Membranes and cytosol were recovered from the high-speed pellet (HSP) and high-speed supernatant (HSS), respectively, and analyzed by SDS gel electrophoresis and western blotting using the indicated antibodies.
C) HeLa cells were mock-treated or treated with two different siRNAs against SNX3 (1 and 2) or against Hrs (Hrs2) for 72 h. Cells were harvested and total lysates were analyzed by SDS gel electrophoresis and western blotting using antibodies against Hrs, SNX3, and actin.
(D) HeLa cells were mock-treated or treated with siRNAs against Hrs for 72 h. GFP-SNX3 was overexpressed during the last 24 h of the Hrs RNAi treatment. Cells were then harvested and total lysates were analyzed by SDS gel electrophoresis and western blotting using antibodies against α-tubulin, Hrs, and SNX3.
(557 KB PDF)

Figure S9 GFP-SNX3 Is Present on Early Endosomes Containing Both Annexin A1 and Annexin A2 (A) HeLa cells expressing mRFP-SNX3 and/or GFP-Anx A2 were fixed [61] and processed for immunofluorescence using the indicated antibodies. Scale bar indicates 10 μm.
(B) HeLa cells were mock-treated or treated with siRNAs against Hrs with or without GFP-SNX3 overexpression (as in Figures 5C and 6), and then incubated with 0.25 μg/ml EGF and 10 μg/ml cycloheximide for the indicated time periods. Cell lysates (100 μg) were analyzed by SDS gel electrophoresis and western blotting with antibodies against EGFR (as in Figures 1A and S7C) or actin.
(1.10 MB PDF)

Figure S10 Model The current view is that, after endocytosis, the ubiquitinated EGF receptor (EGF-R) is sorted into membrane regions of the early endosome that are coated with clathrin and enriched in PtdInsI3P (sorting platforms). Then, EGF-R interacts sequentially with ESCRT I, II, and III complexes and is incorporated into membrane invaginations (in green), giving rise to a forming ECV/MVB [14,37]. The lumenal vesicles are transported to late endosomes and eventually to lysosomes, where they are degraded together with their receptor cargo. Our previous studies show that VSV envelope fuses with ECV/MVB lumenal membranes, thereby delivering the viral capsid to the lumen of these internal vesicles, where it remains hidden [44]. After delivery to late endosomes, back fusion with the limiting membrane ensures capsid delivery to the cytoplasm. The delivery of anthrax toxin lethal factor to the cytoplasm [51] follows a pathway very similar to that of the nucleocapsid (not indicated here for the sake of simplicity).
(A) SNX3 Overexpression. Our observations indicate that SNX3 overexpression causes an accumulation of multivesicular regions on early endosomes and inhibits ECV/MVB detachment from early endosomes (or maturation) and thus interferes with more-distal transport steps. Then, VSV and EGF-R remain in early endosomes and viral RNA release is inhibited. By contrast, Hrs overexpression was reported to mimic its down-expression by RNAi [11,65].
(B) SNX3 RNAi. We find that SNX3 knockdown reduces the formation of ECV/MVB lumenal vesicles, but not EGF-R transport to late endosomes and degradation in lysosomes, indicating that EGF-R sorting can be uncoupled from its incorporation into ECV/MVB lumenal vesicles. Presumably, Hrs (which is not affected by SNX3 knockdown) then ensures proper EGFR targeting to the lysosomes. Consistently, the delivery of viral RNA to the cytoplasm after SNX3 knockdown no longer requires transport to late endosomes, presumably because virions then fuse with the limiting membrane of “empty” ECV/MVBs, devoid of internal vesicles.
(C) Hrs RNAi. Hrs knockdown reduces the formation of ECV/MVB lumenal vesicles [20,29,30] and causes a concomitant decrease in SNX3, which is likely to be responsible for the reduction in lumenal vesicles. Indeed, these can be restored by SNX3 re-expression after Hrs knockdown. After Hrs knockdown, the delivery of viral RNA to the cytoplasm no longer requires transport to late endosomes, because virions then fuse with the limiting membrane of “empty” ECV/MVBs [44]—much like after SNX3 knockdown. In addition, EGF-R transport to late endosomes is not affected, again much like after SNX3 knockdown. These data together with observations from others that EGF-R can be transported towards lysosomes after Hrs knockdown [20] suggest that Hrs functions are dispensable for early-to-late endosomal transport. Then, EGF-R sorting in early endosomes may be achieved by other components of the sorting machinery. Alternatively, a disruption of this machinery may allow uncontrolled EGF-R incorporation into the degradation pathway, including, for example, nonubiquitinated molecules. In any case, and in marked contrast to SNX3 knockdown, we find that EGF-R degradation in lysosomes is seriously compromised by Hrs knockdown, as observed by others [29,30], confirming the crucial function of Hrs for EGF-R lysosomal targeting.
(46 KB PDF)

## Abbreviations

ECV: endosomal carrier vesicle
EGF: epidermal growth factor
GFP: green fluorescent protein
HRP: horseradish peroxidase
LBPA: lysobisphosphatidic acid
mRFP: monomeric red fluorescent protein
MVB: multivesicular body
RNAi: RNA interference
siRNA: small interfering RNA
SNX: sorting nexin
VSV: vesicular stomatitis virus
