# Supplementary material for: Hrs and SNX3 Functions in Sorting and Membrane Invagination within Multivesicular Bodies
Source: PLoS Biol. 2008 Sep 2;6(9):e214. doi: 10.1371/journal.pbio.0060214 (PMC2528051; doi:10.1371/journal.pbio.0060214)

Figure-S2 (Pons)

EGF (A), dextran (B) and Shiga toxin B-subunit (C) reach early endosomes in cells expressing SNX3

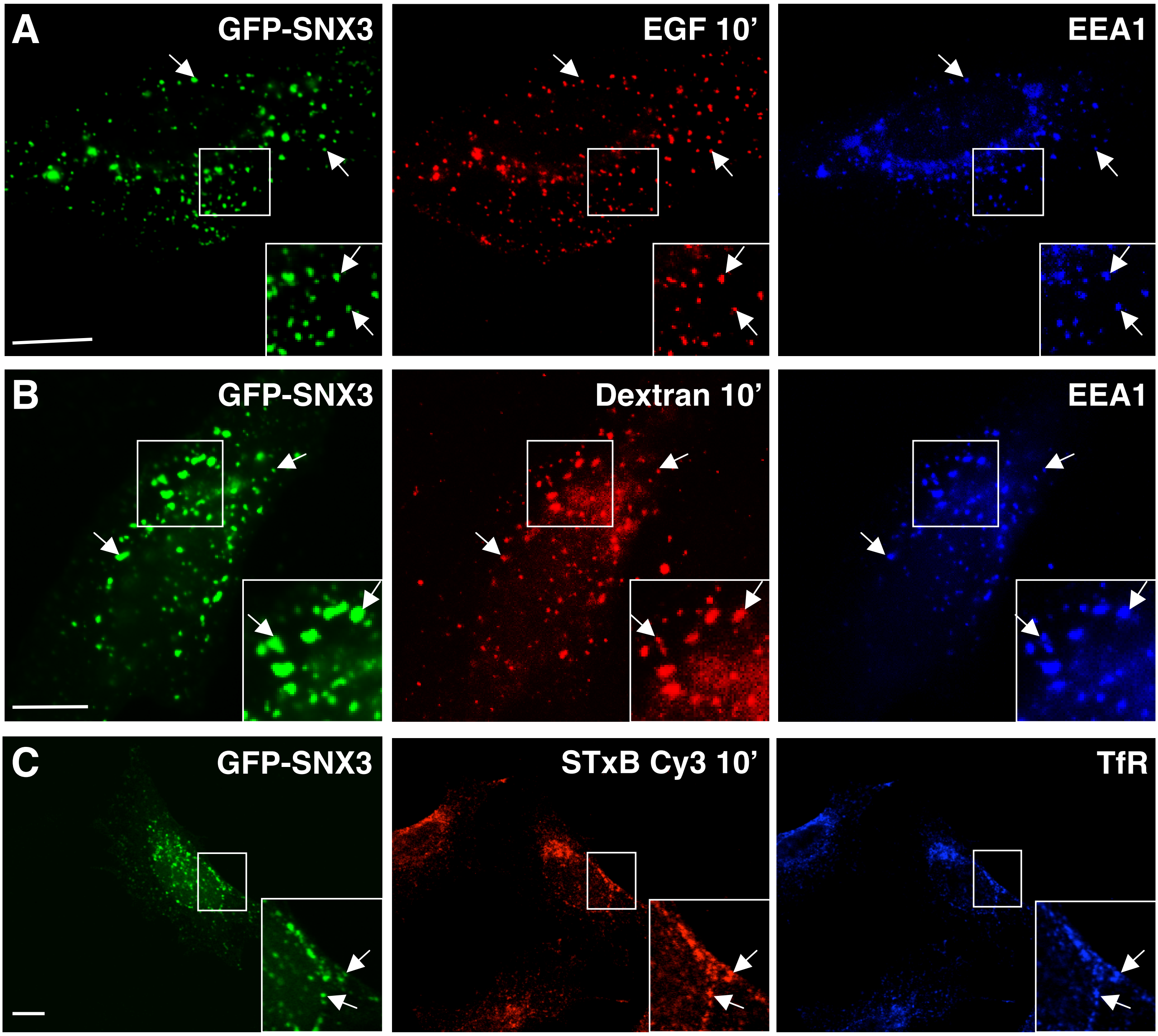

Supplement: Supplementary file 2 [file pbio.0060214.sg002.pdf]
