# Supplementary material for: Hrs and SNX3 Functions in Sorting and Membrane Invagination within Multivesicular Bodies
Source: PLoS Biol. 2008 Sep 2;6(9):e214. doi: 10.1371/journal.pbio.0060214 (PMC2528051; doi:10.1371/journal.pbio.0060214)

# Figure-S3A (Pons)

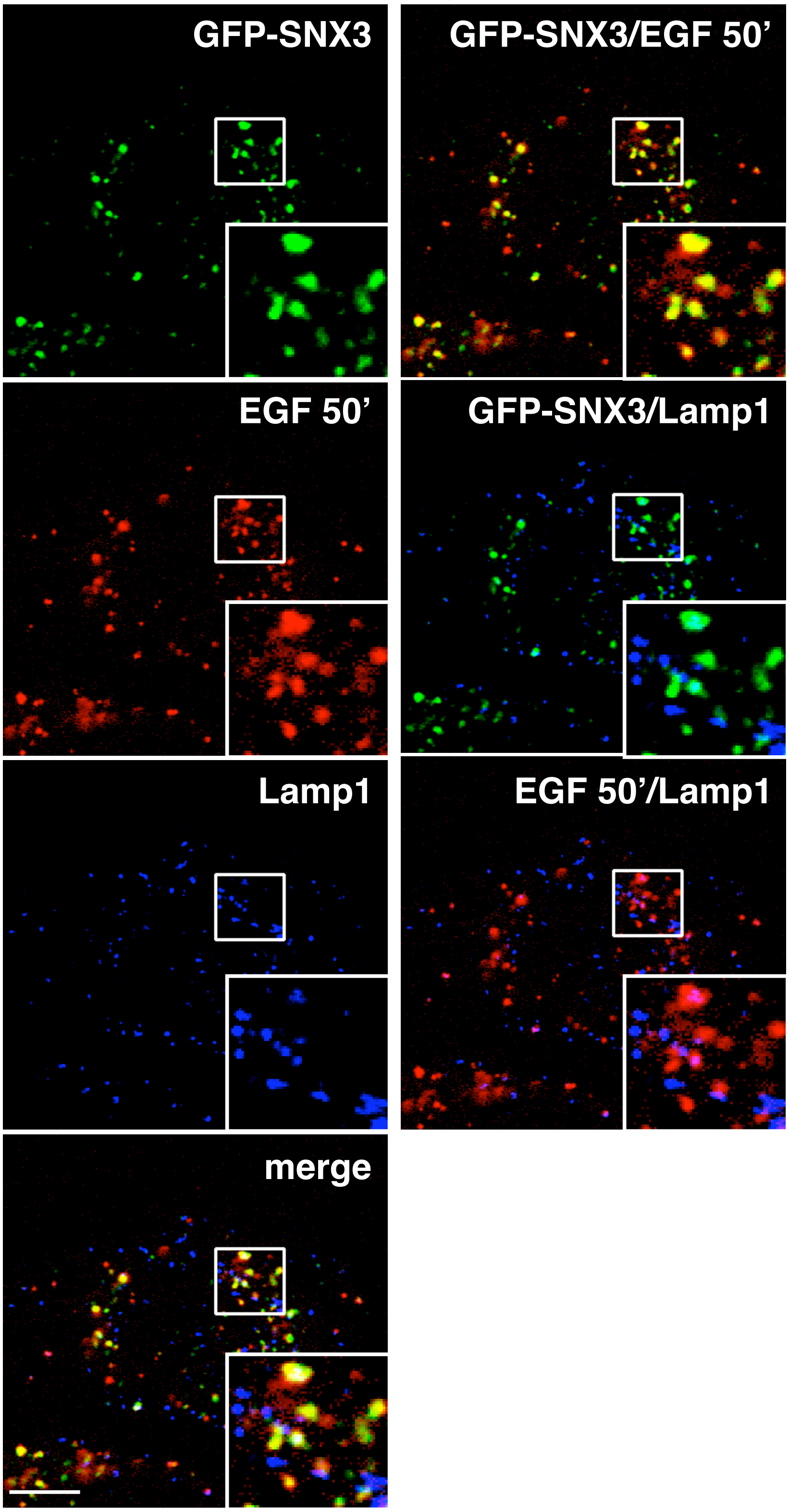

# Figure-S3B (Pons)

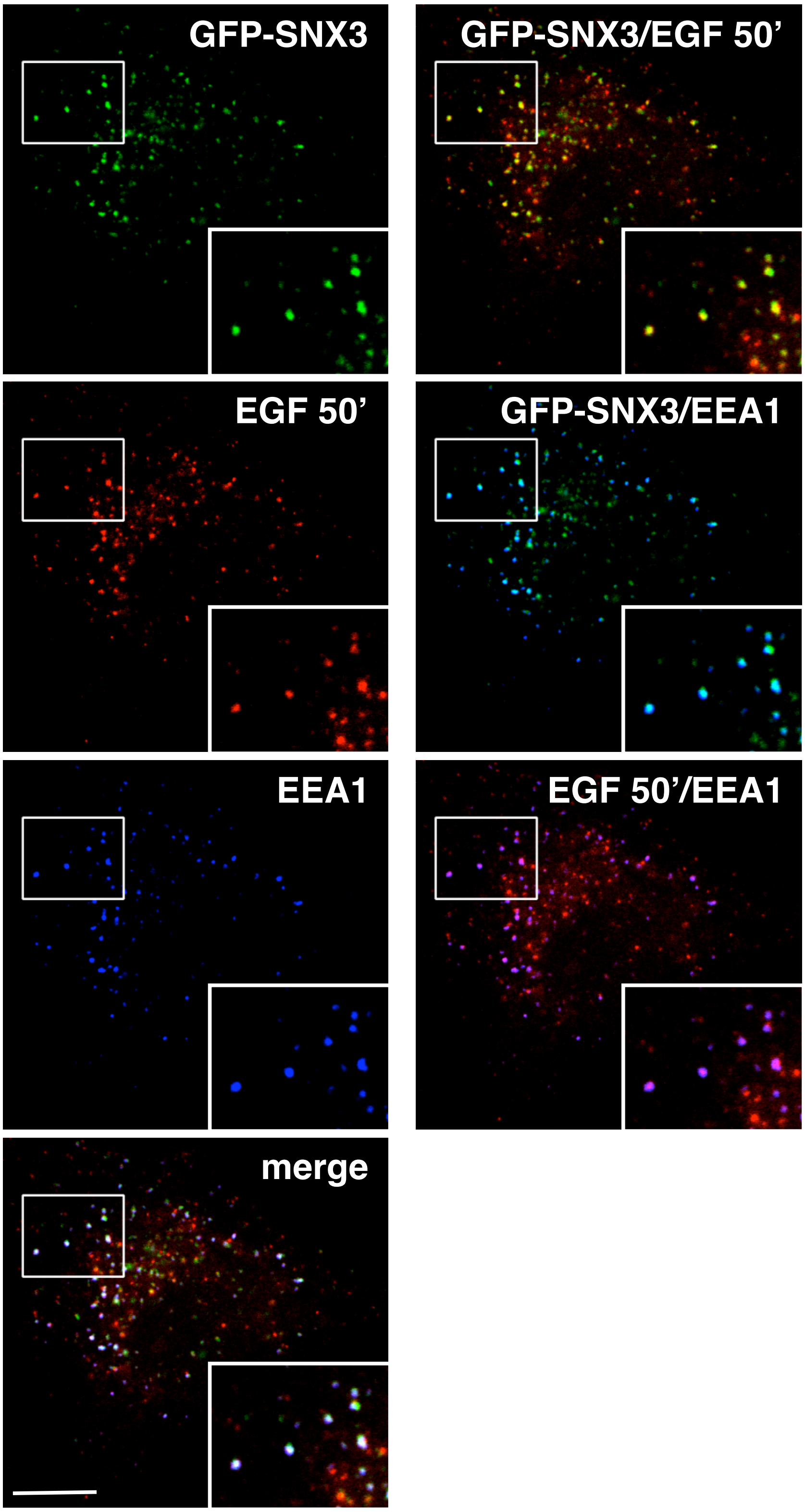

# Figure-S3C (Pons)

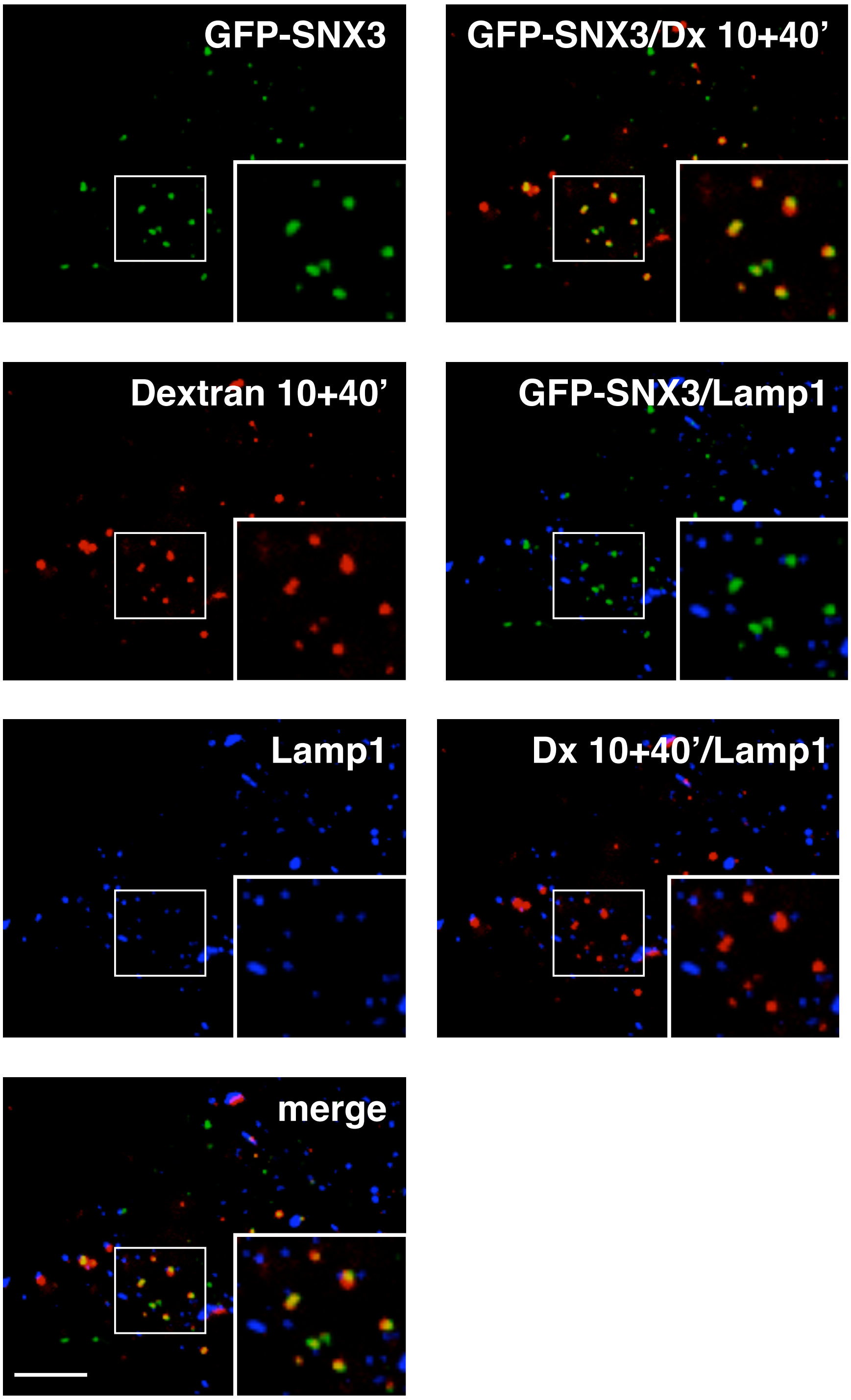

# Figure-S3D (Pons)

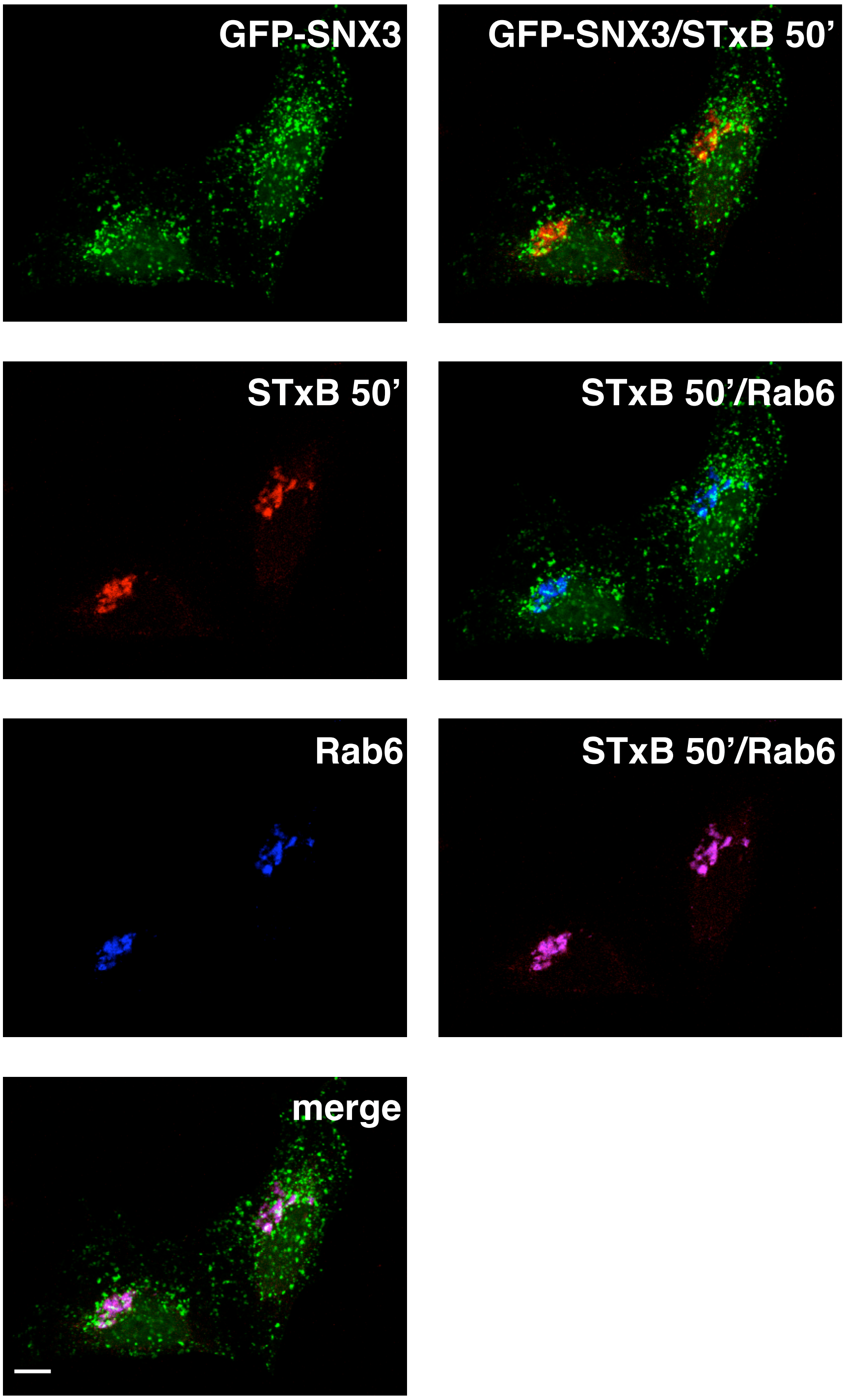

Supplement: Supplementary file 3 [file pbio.0060214.sg003.pdf]
