# Supplementary material for: Hrs and SNX3 Functions in Sorting and Membrane Invagination within Multivesicular Bodies
Source: PLoS Biol. 2008 Sep 2;6(9):e214. doi: 10.1371/journal.pbio.0060214 (PMC2528051; doi:10.1371/journal.pbio.0060214)

Figure-S4 (Pons)

A In control cells, EGF is no longer detected in early endosomes after 50 min

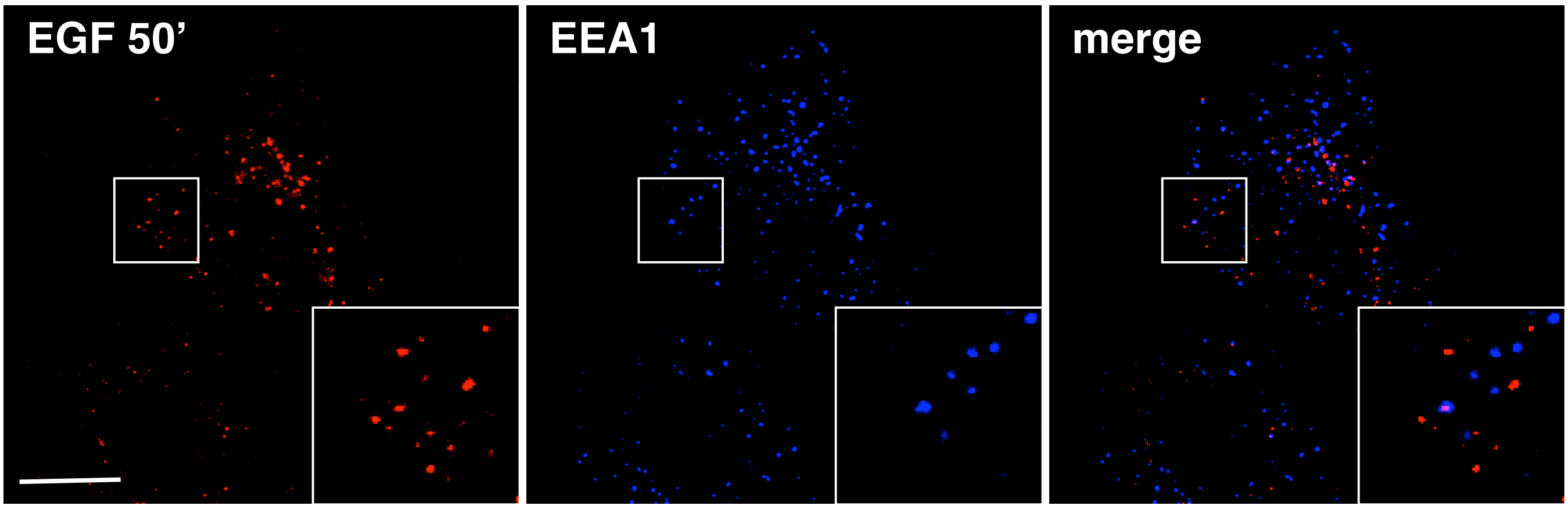

B In control cells, dextran reaches Lamp1-positive late endosomes

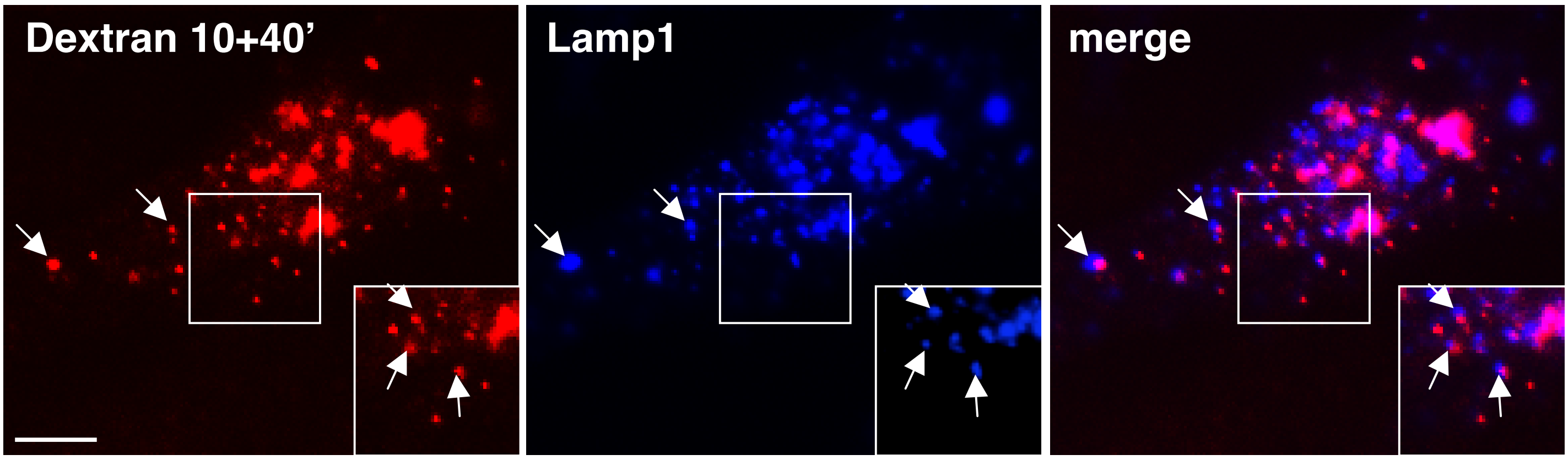

C In control cells, Shiga toxin B-subunit reaches the Rab6-positive Golgi apparatus

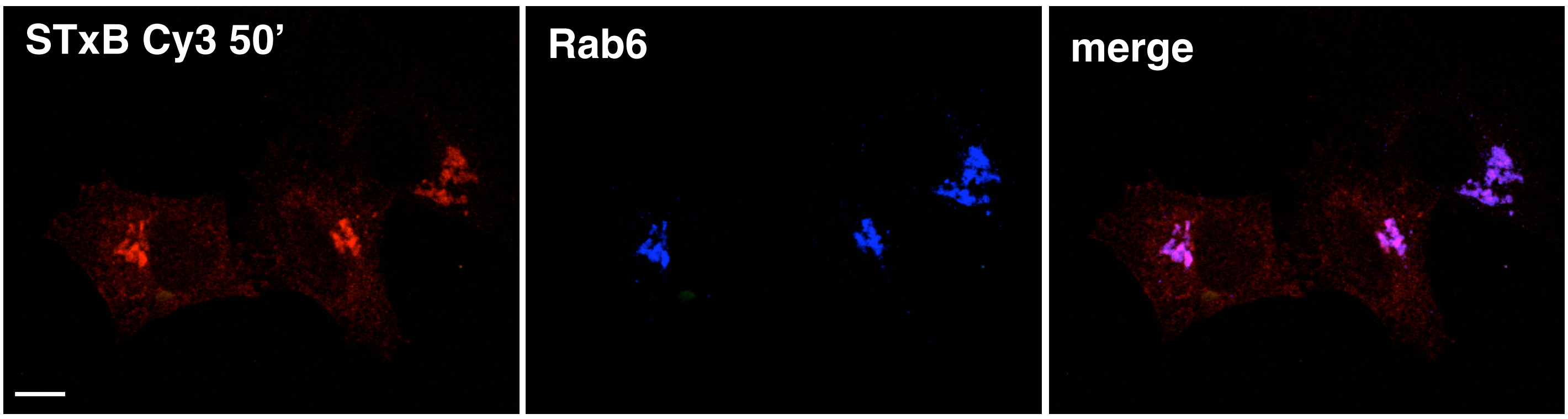

Supplement: Supplementary file 4 [file pbio.0060214.sg004.pdf]
