# Supplementary material for: Hrs and SNX3 Functions in Sorting and Membrane Invagination within Multivesicular Bodies
Source: PLoS Biol. 2008 Sep 2;6(9):e214. doi: 10.1371/journal.pbio.0060214 (PMC2528051; doi:10.1371/journal.pbio.0060214)

Figure-S5 (Pons)

Overexpression of SNX1, SNX2 or SNX 16 does not affect transport of the EGF receptor to the late endosomes and lysosomes

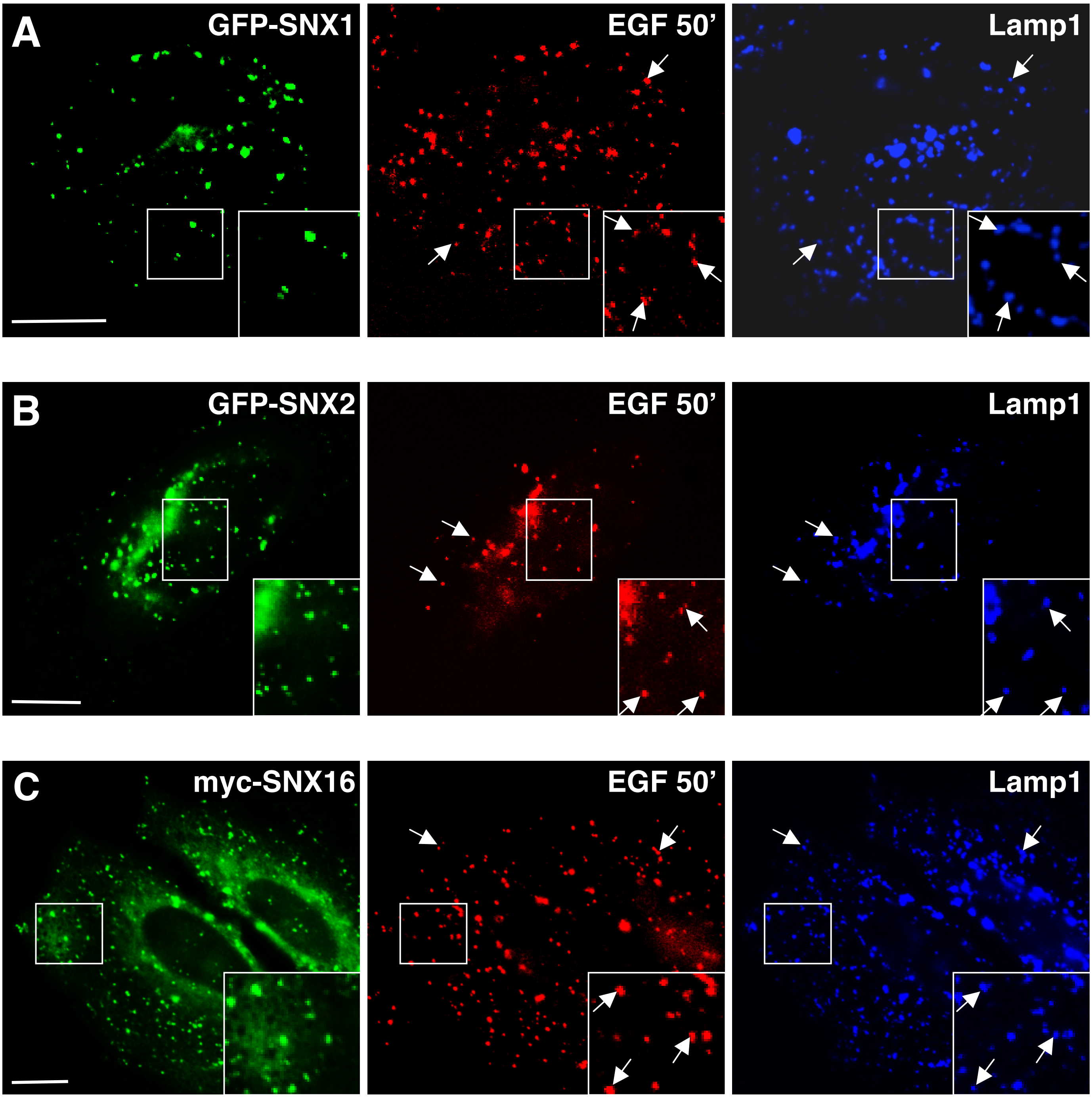

Supplement: Supplementary file 5 [file pbio.0060214.sg005.pdf]
