# Supplementary material for: Hrs and SNX3 Functions in Sorting and Membrane Invagination within Multivesicular Bodies
Source: PLoS Biol. 2008 Sep 2;6(9):e214. doi: 10.1371/journal.pbio.0060214 (PMC2528051; doi:10.1371/journal.pbio.0060214)

Figure-S7 (Pons)

(A-B) EGF transport to late endosomes remains microtubule-dependent in HeLa cells treated with SNX3 siRNA.

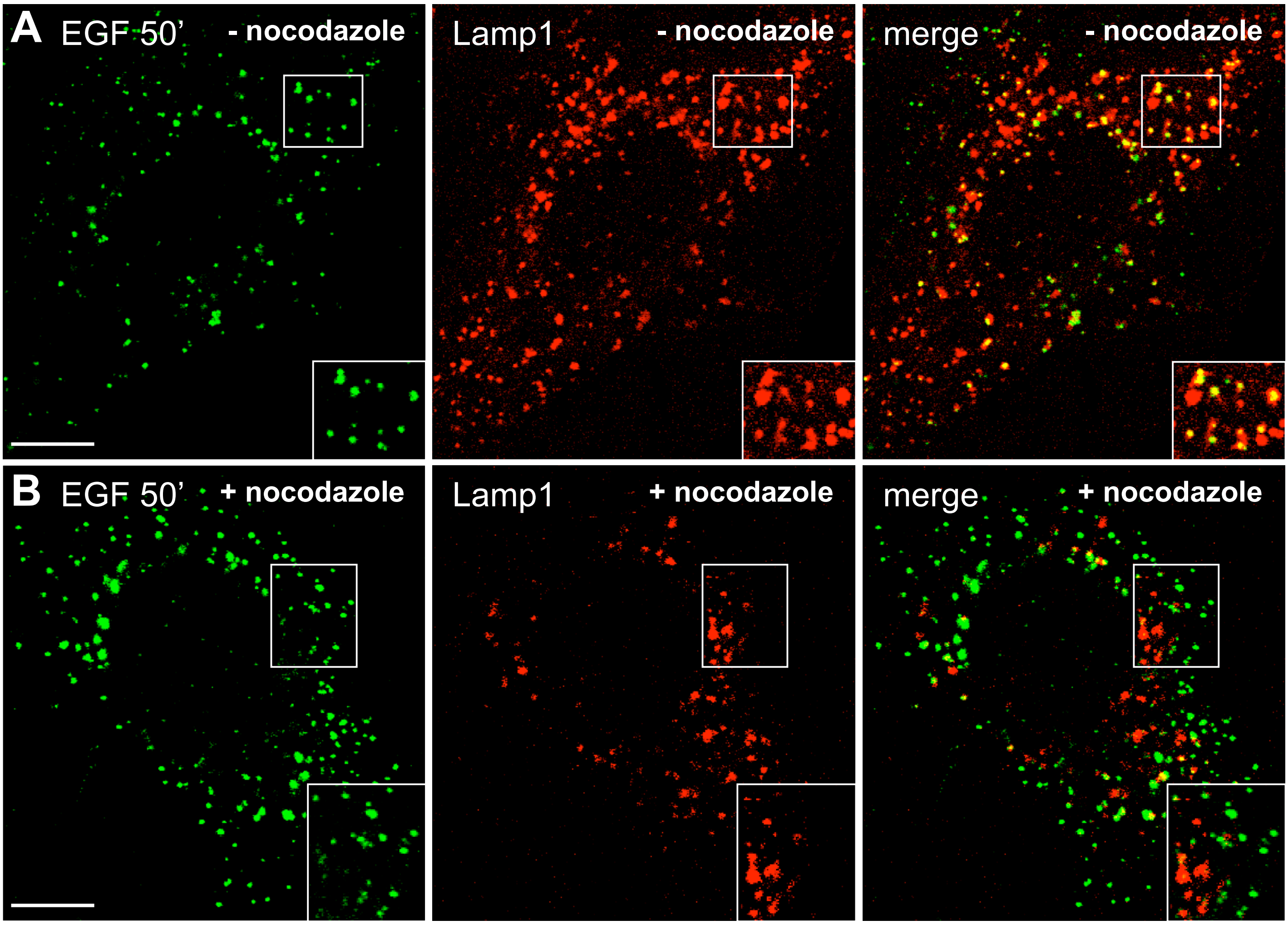

(C) EGF receptor degradation is not significantly affected by SNX3 siRNA

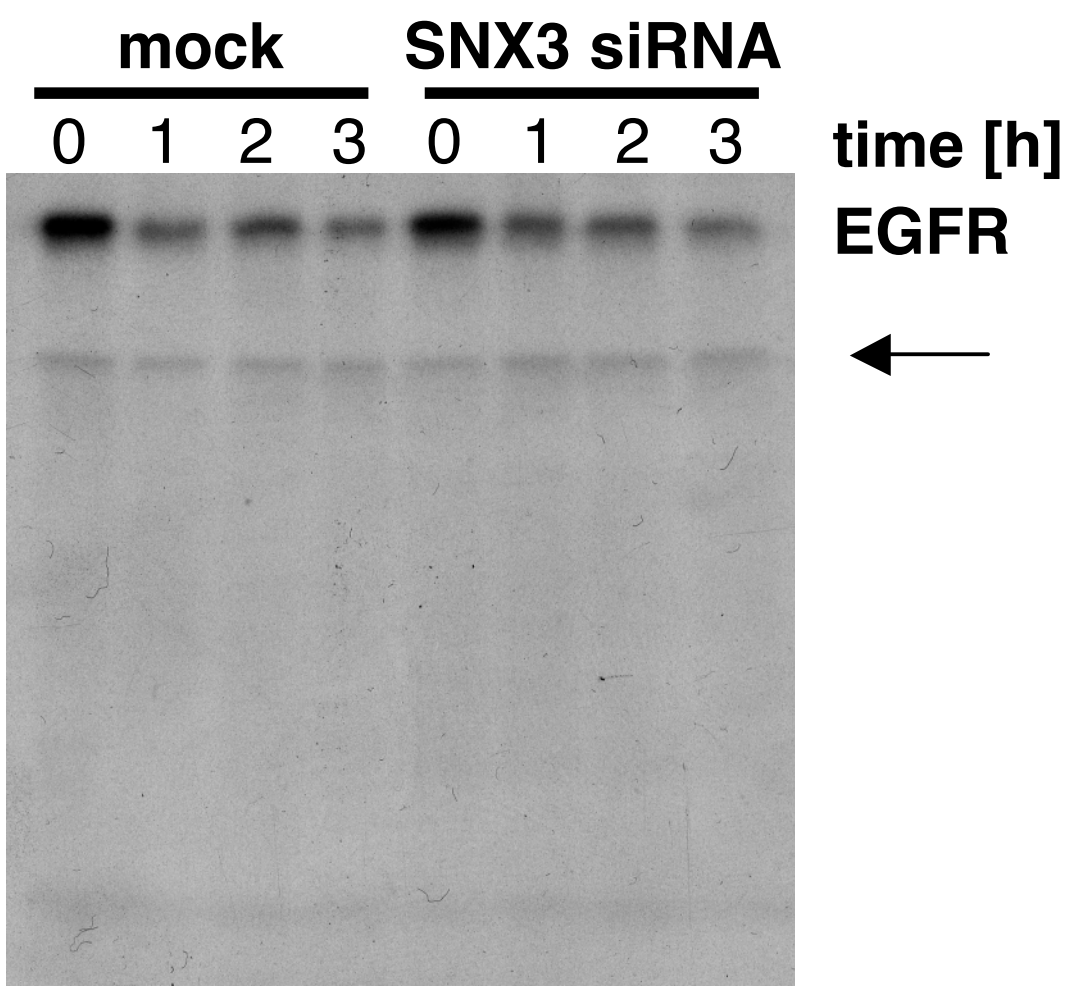

Supplement: Supplementary file 7 [file pbio.0060214.sg007.pdf]
