# Supplementary material for: Hrs and SNX3 Functions in Sorting and Membrane Invagination within Multivesicular Bodies
Source: PLoS Biol. 2008 Sep 2;6(9):e214. doi: 10.1371/journal.pbio.0060214 (PMC2528051; doi:10.1371/journal.pbio.0060214)

Figure-S8 (Pons)

A EGF leaves early endosomes and reaches late endosomes in cells treated with mock siRNA

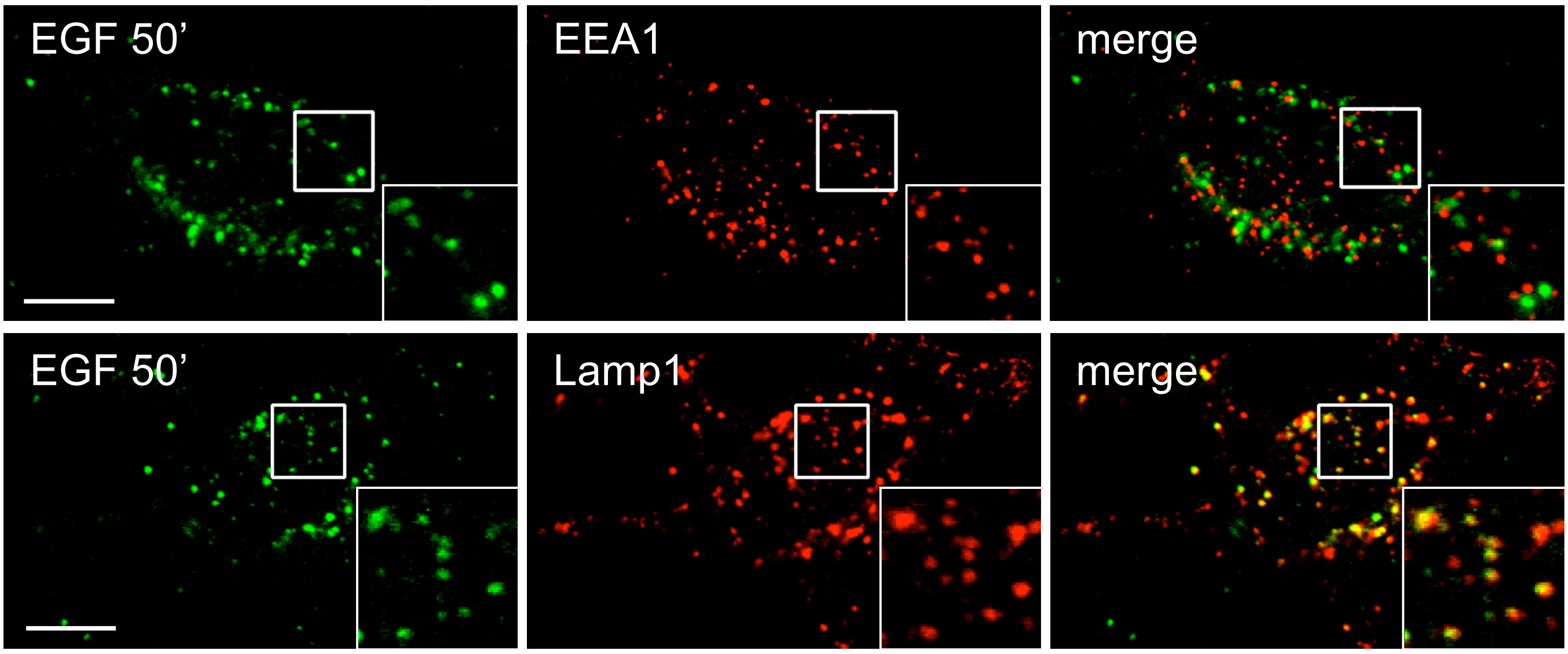

B Hrs siRNA decrease membrane-associated SNX3

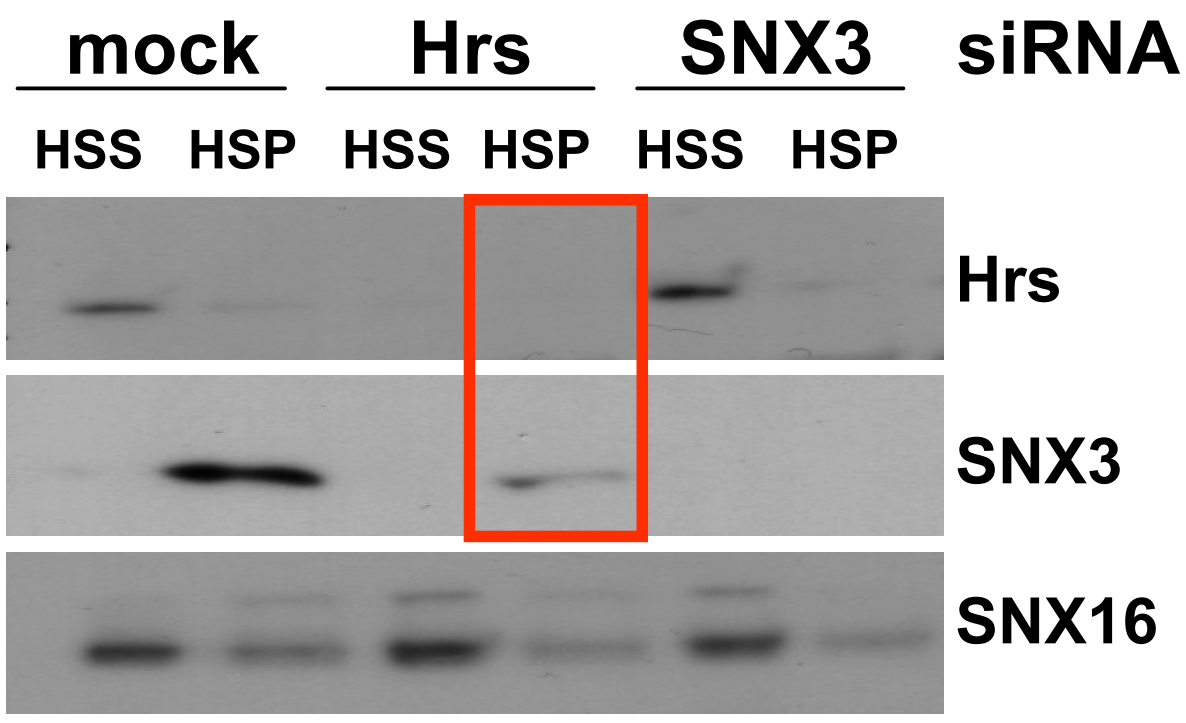

C SNX3 knockdown does not affect Hrs levels

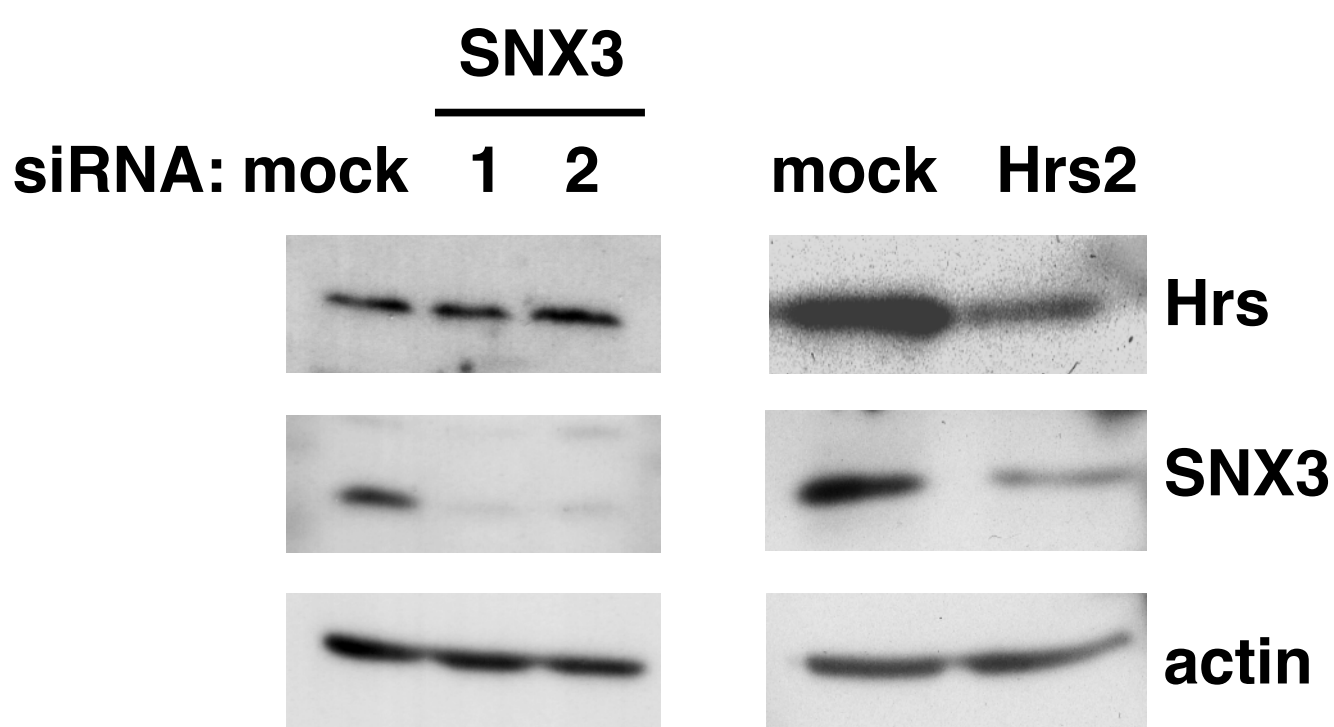

D Re-expression of GFP-SNX3 after Hrs & SNX3 depletion with Hrs siRNA

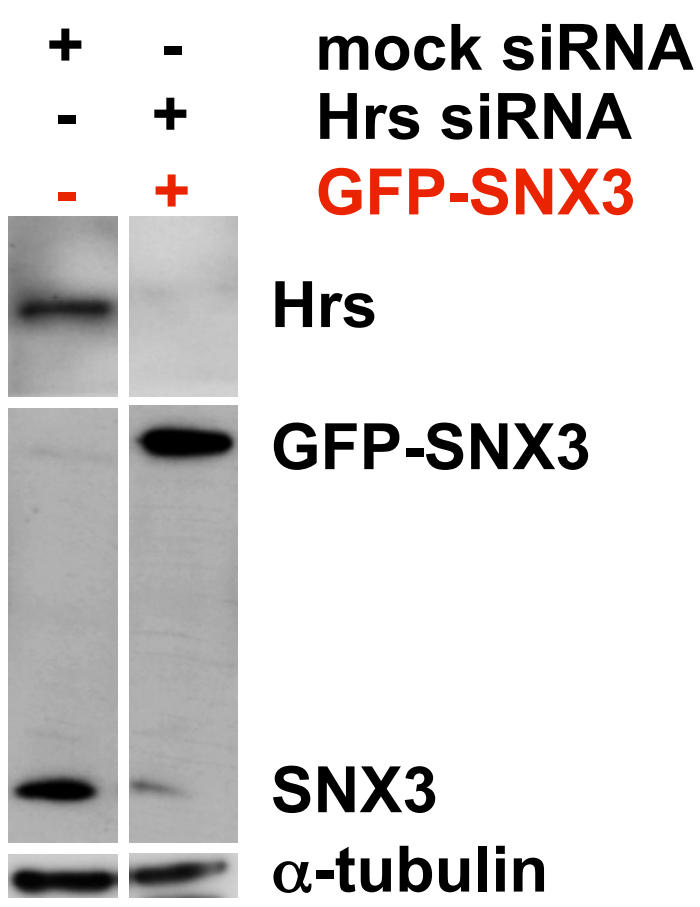

Supplement: Supplementary file 8 [file pbio.0060214.sg008.pdf]
