# Supplementary material for: Hrs and SNX3 Functions in Sorting and Membrane Invagination within Multivesicular Bodies
Source: PLoS Biol. 2008 Sep 2;6(9):e214. doi: 10.1371/journal.pbio.0060214 (PMC2528051; doi:10.1371/journal.pbio.0060214)

Figure-S9 (Pons)

A **SNX3 is present on structures that contain both annnexin A1 and annexin A2**

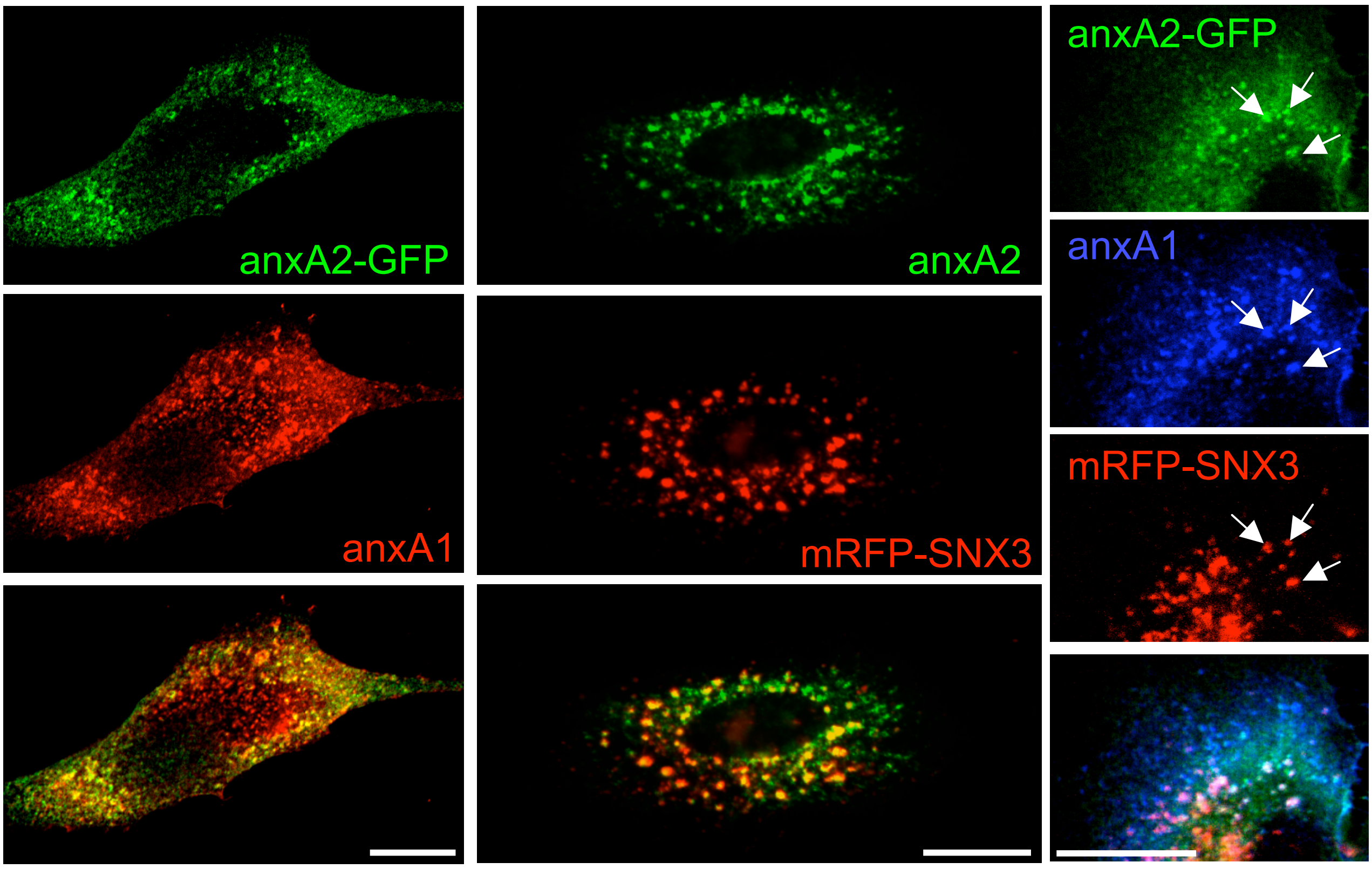

B **EGF-R degradation is inhibited after Hrs depletion and SNX3 re-expression**

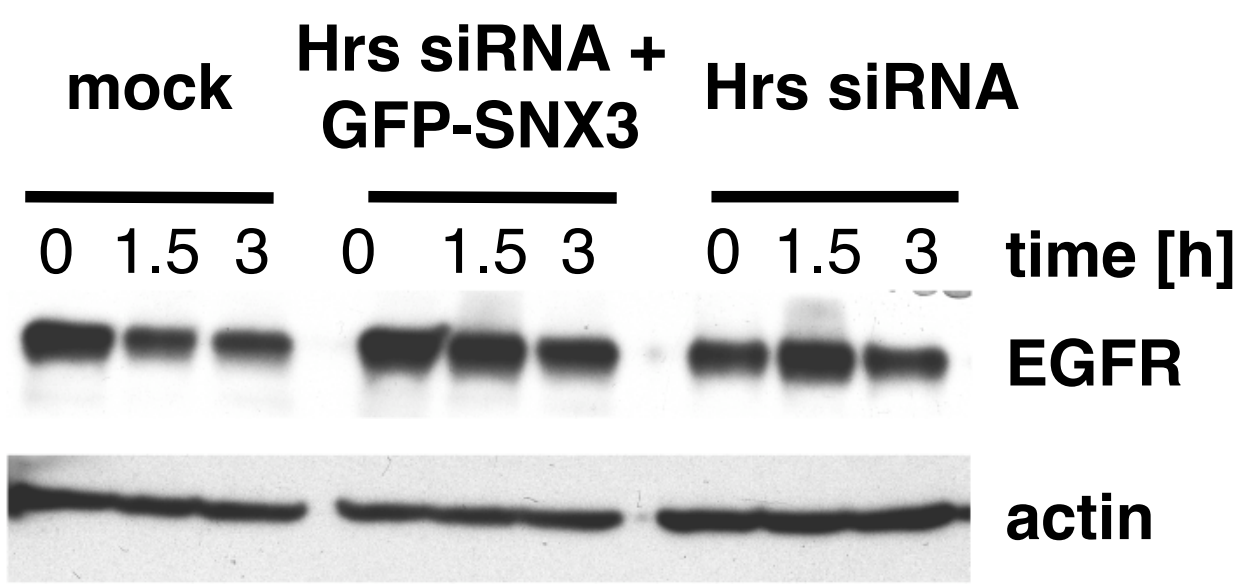

Supplement: Supplementary file 9 [file pbio.0060214.sg009.pdf]
